# A Proteomic Approach to Determine Stem Cell Skeletal Differentiation Signature on Additive Manufactured Scaffolds

**DOI:** 10.1002/smsc.202300316

**Published:** 2024-06-02

**Authors:** Clarissa Tomasina, Ronny Mohren, Sandra Camarero‐Espinosa, Berta Cillero‐Pastor, Lorenzo Moroni

**Affiliations:** ^1^ MERLN Institute for Technology‐inspired Regenerative Medicine Complex Tissue Regeneration Department Maastricht University P.O. Box 616 6200 MD Maastricht The Netherlands; ^2^ The Maastricht MultiModal Molecular Imaging Institute (M4i) Division of Imaging Mass Spectrometry Maastricht University 6200 MD Maastricht The Netherlands; ^3^ POLYMAT Basque Center for Macromolecular Design and Engineering Joxe Mari Korta Center ‐ Avda. Tolosa, 72 20018 Donostia‐San Sebastian Spain; ^4^ IKERBASQUE Basque Foundation for Science 48009 Bilbao Spain

**Keywords:** additive manufacturing, bone, cartilage, mesenchymal stem cells, proteomics

## Abstract

Understanding how porous biomaterials interact with cells at their surface and how they either promote or inhibit cellular processes has presented several challenges. Additive manufacturing enables the fabrication of scaffolds with distinct compositions and designs for different tissue engineering applications. To evaluate the in vitro performance of multiple printed materials, biochemical assays can be limiting in providing valuable insight and key information to select the best tissue destination. Omics technologies like proteomics are crucial for studying important cellular events and gathering valuable information about cellular processes and mechanisms. However, only few studies focus on proteomics to decipher cell–material interactions and cell differentiation on additive manufactured scaffolds. Here, scaffolds were fabricated using three polymers (polycaprolactone (PCL), poly(ethylene oxide)–poly(butylene terephthalate) (PEOT/PBT), and polylactic acid (PLA)) through additive manufacturing. Their chondrogenic and osteogenic potential were characterized and compared using human bone marrow‐derived mesenchymal stem cells (hBMSCs) through proteomics analysis. The 3D scaffolds were all hydrophilic and displayed Young's moduli close to those of bone or cartilage for PLA and PCL and PEOT/PBT, respectively. Biochemical assays indicated that PEOT/PBT and PLA scaffolds have a greater chondrogenic potential by higher glycosaminoglycan (GAG) and collagen deposition compared to PCL. PLA and PEOT/PBT showed to be more effective in promoting bone formation, as evidenced by higher calcium deposits detected by alizarin red staining, and higher alkaline phosphatase (ALP), especially for PLA in osteogenic medium. Proteomics data revealed the most distinct separation between conditions in chondrogenic medium, which had the highest protein identification rates. Pathway analysis showed that PCL did not induce any differentiation‐related pathways when compared to PEOT/PBT and PLA in any of the tested media conditions. Analysis of PEOT/PBT proteins showed pathways involved in chondrogenesis in all three media and pathways related to hypertrophic phenotype progression in chondrogenic medium. These data suggests that PEOT/PBT is a valuable candidate for cartilage and osteochondral applications, able to drive hBMSCs differentiation without the need of growth factors. PLA was also a valuable candidate for cartilage and bone applications by upregulating both chondrogenic and osteogenic‐related proteins in maintenance and chondrogenic media. In osteogenic and maintenance media, the upregulation of angiogenic proteins makes PLA a better candidate for bone application where vascularization is key.

## Graphical abstract

This study investigates the skeletal potential of 3D printed scaffolds made from three biodegradable polymers used in scaffold‐driven tissue regeneration strategies: polycaprolactone, poly(ethylene oxide)–poly(butylene terephthalate) (PEOT/PBT), and polylactic acid (PLA). Human mesenchymal stem cells (hBMSCs) are cultured on these scaffolds, and their differentiation analyzed using proteomics in addition to conventional biochemical assays. PEOT/PBT is promising for cartilage and osteochondral applications, while PLA shows potential for vascularized bone applications.

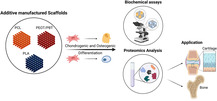

## 1 Introduction

Tissue engineering has achieved extensive progress over the past decades by relying on a wide range of polymeric biomaterials designed to achieve a specific biological effect on cells.^[^ ^1^ ^]^ Among the available biomaterials, synthetic polymers have been widely employed for their processability, mechanical properties, biodegradability, and biocompatibility. Common synthetic polymers with these properties are polyesters such as polycaprolactone (PCL), poly(ethylene oxide terephthalate)/poly‐(butylene terephthalate) (PEOT/PBT), and polylactic acid (PLA).^[^ ^2^ ^]^ Multiple fabrication techniques such as additive manufacturing, gas foaming, solvent casting, and self‐assembly have been employed to shape these materials into 3D scaffolds able to support cells.^[^ ^3^ ^]^ Among the conventional biofabrication methods, additive manufacturing is widely used in tissue engineering because it allows the creation of complex 3D scaffolds with tunable porosity and high reproducibility.^[^ ^4^ ^]^ To investigate the application of additive manufactured materials in tissue engineering applications, their physical, chemical, mechanical, and degradability properties as well as their biological performance need to be investigated.^[^ ^5^ ^]^ In particular, in vitro and in vivo studies focus their attention on the cell–material interactions and offer a preliminary tool to evaluate the cell response of the material as well as its regenerative potential. Furthermore, cell morphology, viability, metabolic activity, proliferation, migration, and differentiation are key factors to evaluate the biological phenomena at the interface. Yet, the conventional molecular biology protocols such as biochemical assays, bioimaging, and genetic and protein tools focus their attention on a limited and selective pool of markers usually characteristics of the target tissue. Although these protocols might be sufficient for a preliminary evaluation of the material performance, they still provide a limited cellular readout that might be not suitable to elucidate the dynamics at the cell‐material interface of a more complex environment such as 3D cultures in scaffolds.^[^ ^6^ ^]^ In fact, in a previous work,^[^ ^7^ ^]^ to compare the biological potential of printed PCL and PLA scaffolds, cell morphology assessment seemed insufficient to elucidate which material could improve osteogenesis in long term cultures. In another study,^[^ ^8^ ^]^ researchers evaluated the differentiation potential of human bone marrow‐derived mesenchymal stem cells (hBMSCs) into the chondrogenic and osteogenic lineages on 3D printed PEOT/PBT scaffolds. Conventional biochemical assay such as GAG assay and ALP revealed no statistical difference among the three cultured media until day 14 (e.g., control, osteogenic, and chondrogenic media). This raised the unanswered question of how the material itself can improve cell osteogenesis and chondrogenesis, as well as, which osteogenic or chondrogenic markers could be upregulated without the use of soluble factors. Taking advantage of omics tools in addition to biochemical assays would allow to study cell‐material interaction and analyse a wide array of markers to compare how materials can trigger cell processes and differentiation.

Omics technologies have been applied to a wide range of research areas including tissue engineering, but have mainly been used to explore fundamental biology questions.^[^ ^9^ ^]^ Hence, their use on 3D scaffolds could provide a deeper insight into crucial cell phenomena in addition to conventional assays.^[^ ^10^, ^11^ ^]^ In this context, proteomics is the evaluation of the proteins produced by a cell type or organism that provides information about the cell function, differentiation and tissue homeostasis.^[^ ^12^ ^]^ Proteins are critical regulators of cell signalling and intracellular communication that directly affect cell survival, phenotype, and gene expression. Label‐free proteomics has been a useful tool to screen and identify multiple biological molecules during cell culture.^[^ ^13^, ^14^ ^]^ Thanks to proteomics, proteins can be identified through their constituent peptides and their level of abundance can be detected.^[^ ^15^ ^]^ This robust and quantitative analysis allows the investigation of complex and large range of proteins, giving insight into the cell biological systems as well as further enriching the array of biochemical markers to better understand cell culture and differentiation. To evaluate lineage commitment of hBMSCs, Granéli et al.^[^ ^16^ ^]^ used proteomics to identify novel surface markers of cells undergoing osteogenesis, thus unravelling crystalline‐αB (CRYaB) as a new specific marker for early and late stages of osteogenic differentiation.

Among the cells used in tissue engineering, hBMSCs are multipotent stem cells located in the bone marrow and are capable to self‐renew and differentiate into various tissues such as fat, bone, cartilage, and muscle.^[^ ^17^ ^]^ In the context of the skeletal system, hBMSCs have been differentiated toward chondrogenic and osteogenic phenotypes on additive manufactured scaffolds based on different synthetic materials. In a study by Kong et al.^[^ ^18^ ^]^ proteomics was used to explore the effect of a polydopamine coating onto additively manufactured Ti6Al4V scaffolds and correlated its enhanced osteogenic effect with the BMP‐Smads signaling pathway. In another study,^[^ ^19^ ^]^ proteomics analysis helped associating the osteogenic potential of zinc particles incorporated into 3D printed poly (lactic‐co‐glycolic acid)/β‐tricalcium phosphate scaffolds to the activation of the Wnt/β‐catenin, p38 MAPK and the inhibition of the NFkB signaling pathways on BMSCs. Hence, with the aid of proteomics, it is possible to identify the key modulators of hBMSCs proliferation and differentiation on 3D scaffold culture conditions as a function of the biomaterial.

In this study, we conducted a comprehensive investigation to determine which additive manufacturing biomaterial scaffold—PCL, PEOT/PBT, or PLA—is optimal for bone and cartilage applications. To this end, we performed proteomics characterization along with traditional biochemical assays on hBMSCs cultured in osteogenic, chondrogenic, and maintenance media. Our analysis focused on the difference of the cell proteome in relation to the material used. At the end of the differentiation, pathway analysis gave us a deep insight on how hBMSCs differentiate on PCL, PEOT/PBT, and PLA 3D scaffolds and which material would be more suitable for bone or cartilage applications.

## 2 Experimental Section

Polycaprolactone (PCL, Mn 45 000) and polylactic acid (PLA, Natureworks 4052D) were purchased from Sigma Aldrich and NatureWorks, respectively. Poly(ethylene oxide terephthalate)/poly‐(butylene terephthalate) (PEOT/PBT block copolymer, Polyactive) was kindly provided by Polyvation B.V. with a weight ratio of PEOT/PBT of 55/45 and a molecular weight (g/mol) of the starting PEG segments used in the polymerization process of 300.

### 2.1 Additive Manufacturing

Scaffolds were fabricated using a Bioscaffolder system (SYSENG) equipped with a temperature controller and a G25 needle (Ø: 0.250 mm). The three materials were fabricated using different parameters but targeting the same fiber spacing, layer thickness and angle of deposition of 550 μm, 200 μm, and 90°, respectively. PCL was deposited at a temperature of 95 °C using a travel speed of 500 mm min^−1^ a dispensing speed and pressure of 90 RPM and 0.8 MPa, respectively. PEOT/PBT was dispensed at 195 °C at a travel speed of 400 mm min^−1^, dispensing speed of 60 RPM and dispensing pressure of 1.0 MPa. PLA was deposited at 180 °C using a travel speed of 500 mm min^−1^, a dispensing speed of 70 RRPM and a dispensing pressure of 1.0 MPa.

The scaffolds printing error was evaluated using the following equation:

(1) ε=(d fiber−d needle)/d fiber

where, *d* is diameter and *ε* is the error. The diameter was calculated from scanning electron microscopy (SEM) images.

### 2.2 GPC

Samples of the three materials before and after printing were dissolved in chloroform at a concentration of 2 mg mL^−1^ and filtered through a 0.2 μm PTFE pore diameter filter. The average number and mass molecular weights (Mn and Mw, respectively) and the polydispersity index (Mn/Mw, PI) were measured with a Shimadzu LC‐2030 Performance‐i gel permeation chromatography (GPC) equipped with an RI detector RID‐20A. The column used was a Shodex KF‐850L and chloroform was used as eluent from Carl Roth (HPLC quality). The GPC column was calibrated with a polystyrene standard up to 200.000 g mol^−1^.

### 2.3 Surface Wettability

Polymer films of PCL, PEOT/PBTPBT, or PLA were created using a manual Specac hot‐press. Polymer pellets were placed between silicon wafers at a temperature of 95, 195, and 180 °C applied for PCL, PEOT/PBT, and PLA, respectively. Polymer films sandwiched between silicon wafers were then quenched using running tap water.

After the film fabrication, the water contact angle (WCA) was measured using a goniometer system (DSA4) equipped with an electronic syringe and a CCD camera. On the surface of each film, a sessile drop of 4 μL Milli‐Q water was placed at room temperature and the angle between the tangent of the drop and the substrate was measured from the acquired images. A total of three droplets were deposited on each film.

### 2.4 Mechanical Testing

Cubical Scaffolds of 4 × 4 × 4 mm were tested under compression. First, samples were measured with a caliper and then tested on a TA ElectroForce (TA Instruments) equipped with a 450 N load cell in dry state. Second, scaffolds were compressed at a strain rate of 0.01 mm s^−1^ until 70% deformation. The data was recorded with Wint7 software and was then analyzed from strain–stress curves. Finally, the Young's modulus was calculated from the slope of the strain–stress curve between 0.2 and 1.2 strain.

### 2.5 BMSCs Culture

HBMSCs isolated from bone marrow were purchased from Texas A&M Health Science Center, College of Medicine, Institute for Regenerative Medicine (Donor 803.1 L) and were subcultured at around 80% until passage 5. BMSCs were expanded at 1000 cells cm^−2^ and cultured in basal media composed of minimum essential media (α‐MEM, Gibco) with 10% fetal bovine serum (FBS) (Sigma).

### 2.6 BMSCs Culture on Additive Manufactured Scaffolds

Additive manufactured scaffolds were cut in cubes of 4 × 4 × 4 mm dimensions and then disinfected for 15 min in 70% ethanol, dried on filtered paper and finally placed in 24 well plates. BMSCs at passage 5 were seeded at 250 000 cells/per sample and incubated for 7 days at 37 °C and 5% CO_2_ in basal medium supplemented with 100 U mL^−1^ penicillin‐streptomycin (Sigma). Medium was refreshed every second day. After 7 days of culture, medium was changed to chondrogenic or osteogenic medium for 3 weeks. Chondrogenic medium was composed by high glucose (4.5 mg mL^−1^) DMEM (Thermo Fisher) supplemented with 10 μg mL^−1^ sodium pyruvate, 100 U mL^−1^ penicillin‐streptomycin, 100 μg mL^−1^ insulin‐transferrin‐selenium (ITS) liquid media supplement (Thermo Fischer Scientific), 0.2 mM ascorbate‐2‐phosphate (Sigma‐Aldrich), 100 nM dexamethasone (Sigma), 40 μg mL^−1^ proline (Sigma Aldrich) and 0.01 μg mL^−1^ TGF‐β3 (dissolved according to manufacture instructions, Peprotech). Osteogenic media was comprised of α‐MEM supplemented with 10% FBS, 100 U mL^−1^ penicillin‐streptomycin, 10 nM dexamethasone, 50 μM ascorbate‐2‐phosphate, and 10 mM β‐glycerol phosphate disodium salt (Sigma Aldrich). Samples in basal media were cultured as controls. Media was changed three times a week. During the cell culture studies, the following abbreviation were used to identify the samples and will be used to discuss the results in this work (**Table** **1**).

**Table 1:** Sample nomenclature.

Material	Basic Media	Chondrogenic Media	Osteogenic Media
Polycaprolactone (PCL)	PCL_M	PCL_C	PCL_O
Polyactive45/55 (PA)	PEOT/PBT_M	PEOT/PBT_C	PEOT/PBT_O
Polylactic acid (PLA)	PLA_M	PLA_C	PLA_O

### 2.7 DNA Quantification

Samples were washed with phosphate saline buffer (PBS) and placed dry in 2 mL Eppendorf tubes. The scaffolds were freeze‐thawed three times in liquid nitrogen and 56 °C to break the ECM and later incubated overnight in 250 μL of 1 mg mL^−1^ Proteinase K and 10 μg mL^−1^ Pepstatin A in 50 mM TRIS/1Mm ethylenediaminetetraacetic acid (EDTA) at 56 °C to digest the ECM. Afterwards, samples were freeze‐thawing again three times. Digested samples were then incubated in 1:500 RNAse in lysis buffer for 1 h at room temperature (RT) according to manufactures instructions in the Cyquant cell proliferation assay kit (Thermo Fisher). Finally, 100 μL of each sample in triplicate and 100 μL of 2×GR‐dye were loaded in a 96 well plate with clear bottom and incubated for 10 min in the dark at RT. A standard curve was prepared using DNA standard with the addition of GR‐dye according to the kit's instructions. Fluorescence intensity was measured at 520 nm (emission at 480 nm) using a Clariostat Plate reader.

### 2.8 GAGs Quantification

Samples from the DNA assay after the RNAse incubation step were used for the glycosaminoglycan (GAG) assay. A 1,9‐dimethyl‐methylene blue zinc chloride double salt solution (DMMB) was prepared by dissolving 16 mg of DMMB, 2.37 g NaCl and 3.04 g glycine in 1.0 L of MilliQ water (ph 3.0). In a 96 well plate, 25 μL of samples in triplicate, 5 μL of 2.3 M of NaCl and 150 μL of DMMB were mixed, and absorbance difference was directly measured at 525 and 595 nm using a Clariostat Plate reader. A standard curve was prepared using shark chondroitin sulfate (Sigma‐Aldrich).

### 2.9 ALP Quantification

Samples were washed with PBS and placed in 2 mL Eppendorf tubes. After three freeze‐thaw cycles in liquid nitrogen, samples were incubated 1 h in 250 μL of lysis buffer containing 0.1 M KH_2_PO_4_, 0.1 M K_2_HPO_4_, 0.1% Triton X‐100 in MilliQ water (ph 7.8) at RT. First, 50 μL of samples were transferred to a new Eppendorf while the remaining 200 μL were used to perform the DNA assay (see DNA quantification section). Second, 10 μL of the lysate were loaded in a white bottom 96 well plate together with 40 μL of “CDP‐star‐ready to use” reagent to each well. Finally, the plate was incubated 10 min in the dark at RT before measuring luminescence at 466 nm using a Clariostat Plate reader.

### 2.10 Safranin‐O Staining

Scaffolds were cut in half using a blade prior analysis. Samples were placed in a 24 well plate and fixed for 30 min with 500 μL 4% paraformaldehyde (PFA) and then rinsed 3 times with PBS. First, scaffolds were incubated for 10 min with 500 μL of Weigert's iron hematoxylin (Sigma‐Aldrich) solution to stain the cell nucleus and then rinsed with deionized water three times. Samples were then immersed for 3 min in 500 μL of Fast Green solution (Sigma‐Aldrich) and rinsed by quickly dipping in 0.5% acetic acid solution. Lastly, scaffolds were incubated 5 min with 500 μL of 0.1% Safranin‐O solution (Sigma‐Aldrich) and then rinsed with PBS. Samples were imaged using a Nikon Stereomicroscope SMZ25.

### 2.11 Alizarin Red Staining

Samples were cut in half, placed in a 24 well plate, fixed for 30 min with 500 μL 4% PFA, and then rinsed with PBS three times. An Alizarin Red S solution (ARS) was prepared by dissolving 0.7 g ARS (Sigma) in 50 mL deionized H_2_O (pH 4.1‐4.3). Scaffolds were then washed with deionized H_2_O and submerged for 20 min in 500 μL of ARS solution. Then, scaffolds were rinsed with deionized water until solution was clear. Samples were imaged using a Nikon Stereomicroscope SMZ25.

### 2.12 Immunofluorescence

Samples were cut in half using a scalpel and placed in a 96 well plate. First, samples were fixed for 30 min in 500 μL 4% PFA and then rinsed three times in PBS. Second, samples were permeabilized for 15 min using 500 μL 0.1% Triton X‐100 solution (Sigma) in PBS and then blocked for 1 h in a solution of 3% BSA (Bovine Serum Albumin, Sigma) in 0.01% Triton X‐100 in PBS. Afterwards, samples were rinsed and kept overnight at 4 °C with mouse anti‐collagen I (1:500) (ab90395, Abcam), and rabbit anti‐collagen II (1:400) antibodies (ab34712, Abcam) for chondrogenesis, and mouse anti‐collagen I (1:500) (ab90395, Abcam) and rabbit anti‐osteocalcin (1:400) (ab93876, Abcam) for osteogenesis. AlexaFluor 488 Phalloidin (Thermo Fisher Scientific) was included in both conditions (1:200). After overnight incubation samples were rinsed with a solution of 0.3% BSA, 0.001% Triton X‐100 in PBS. Samples were then incubated with secondary antibodies anti‐mouse AlexaFluor 647 and anti‐rabbit AlexaFluor 568 in the dark for 30 min at RT in PBS (1:200, Thermo Fisher Scientific), followed by staining with Hoechst 33342 (1:1000) in PBS for 10 min and final rinsing with PBS. Samples were stored at 4 °C until imaged using a Leica TCS SP8 CARS confocal microscope.

### 2.13 SEM

Scaffolds were cut after additive manufacturing onto 4 × 4 × 4 blocks and mounted on the support with the use of double‐sided carbon tape. Mounted samples were then coated with gold using a Cressington Sputter coater 108 auto. A FEI/Philipps SEM XL‐30 microscope was used on a secondary electron mode at 10 keV.

To analyze the ECM morphology after culture, samples from immunofluorescence were further analyzed under SEM. Samples that were on PBS after fixation were further incubated on a series of ethanol dilutions (30%, 50%, 70%, 80%, 90%, 96% (1×) and 100% (2×)) for 15 min each at RT. Afterwards, samples were cross‐linked by incubating first in 50:50 ethanol:hexamethyldisilazane (HDMS), then in 100% HDMS for 15 min each, and allowed to dry overnight at RT. Samples were gold coated using a Cressington Sputter coater 108 auto and imaged under a FEI/Philipps SEM XL‐30 microscope at 7 keV.

### 2.14 Label Free Proteomics

Sample preparation and liquid chromatography/mass spectrometry was performed as previously described.^[^ ^20^, ^21^ ^]^

Ammonium bicarbonate, dithiothreitol, iodoacetamide, and trifluoroacetic acid (ULC grade) where purchased from Sigma‐Aldrich, Urea from GE Healthcare and the enzyme mix trypsin/lysC (mass spec grade) was purchased from Promega. Water, acetonitrile, formic acid, all ULC grade, were purchased from Biosolve.

#### 2.14.1 Sample Preparation

##### Protein Isolation

Samples (cells in scaffolds) were collected in 5 M Urea, 50 mM ammonium bicarbonate (ABC) in 1.5 mL Eppendorf tubes. Cell lysis was performed by three freeze‐thaw cycles, using liquid nitrogen for freezing and approximately 40 s of sonicating in an ultrasonic bath for thawing. Proteins in solution were transferred to new tubes, concentrated and desalted using 3 kDa MWCO filters.

##### In Solution Digestion

Protein samples were reduced with 20 mM Dithiothreitol (DTT) for 45 min and alkylated with 40 mM Iodoacetamide (IAM) for 45 min in the darkness. The alkylation was terminated by 20 mM DDT to consume any excess IAM. Digestion was performed with a mixture of LysC and Trypsin, which was added at a ratio of 1:25 (enzyme to protein). After 2 h of digestion at 37 °C in a water bath, the lysate was diluted with 50 mM ABC to 1 M Urea and further digested at 37 °C overnight. The digestion was terminated by addition of formic acid (FA) to a total of 1%.

#### 2.14.2 Liquid Chromatography – Mass Spectrometry

Peptide separation was performed on a Thermo Scientific (Dionex) Ultimate 3000 Rapid Separation UHPLC system equipped with a PepSep C18 analytical column (15 cm, ID 75 μm, 1.9 μm Reprosil, 120 Å). Peptide samples were first desalted on an online installed C18 trapping column. After desalting peptides were separated on the analytical column with a 90 min linear gradient from 5% to 35% Acetonitrile (ACN) with 0.1% FA at 300 nL min^−1^ flow rate. The UHPLC system was coupled to a Q Exactive HF mass spectrometer (Thermo Scientific). DDA settings were as follows. Full MS scan between 350 and 1650 m z^−1^ at resolution of 120 000 followed by MS/MS scans of the top 15 most intense ions at a resolution of 15 000.

#### 2.14.3 Mass Spectrometry Data Analysis

For protein identification and quantitation, DDA spectra were analyzed with Proteome Discoverer (PD) version 2.2. Within the PD software, the search engine Sequest was used with the SwissProt human protein database (Homo Sapiens, SwissProt TaxID = 9606). The database search was performed with the following settings. Enzyme was trypsin, a maximum of 2 missed cleavages, minimum peptide length of 6, precursor mass tolerance of 10 ppm, fragment mass tolerance of 0.02 Da, dynamic modifications of methionine oxidation and protein N‐terminus acetylation, static modification of cysteine carbamidomethylation.

Protein quantitation was performed by using default LFQ (Label Free Quantitation) settings in Proteome Discoverer 2.2. In short, for peptide abundancies the peptide precursor intensities were used and normalization was performed on total peptide amount. Protein ratios were calculated based on pairwise peptide ratios and background based ANOVA was used for hypothesis testing. Pathway Enrichment analysis was performed using String (version 11.5, https://string‐db.org/) with medium confidence for minimum required interaction score and FDR stringency of 5% using Gene Ontology Pathways.

### 2.15 Statistical Analysis

All samples were run in triplicate, *n* = 3. Statistical significance was calculated by two‐way ANOVA, (∗∗∗∗) *p* < 0.0001, (∗∗∗) *p* < 0.001, (∗∗) *p* < 0.01, and (∗) *p* < 0.05. For PCA plots and LC‐MS, duplicate runs were performed for each samples triplicate.

## 3 Results and Discussion

### 3.1 Scaffold Fabrication and Characterization

PCL, PEOT/PBT, and PLA were used separately to fabricate additive manufactured scaffolds with the same structure. After optimizing the processing parameters, the polymers were printed into 20 × 20 × 4 mm squares with fiber spacing of 550 μm, layer‐thickness of 200 μm and printing angle of 90°. This architecture has been proven to allow a compact organization of the ECM as well as cell infiltration and hBMSC differentiation.^[^ ^22^ ^]^

**Figure 1:**
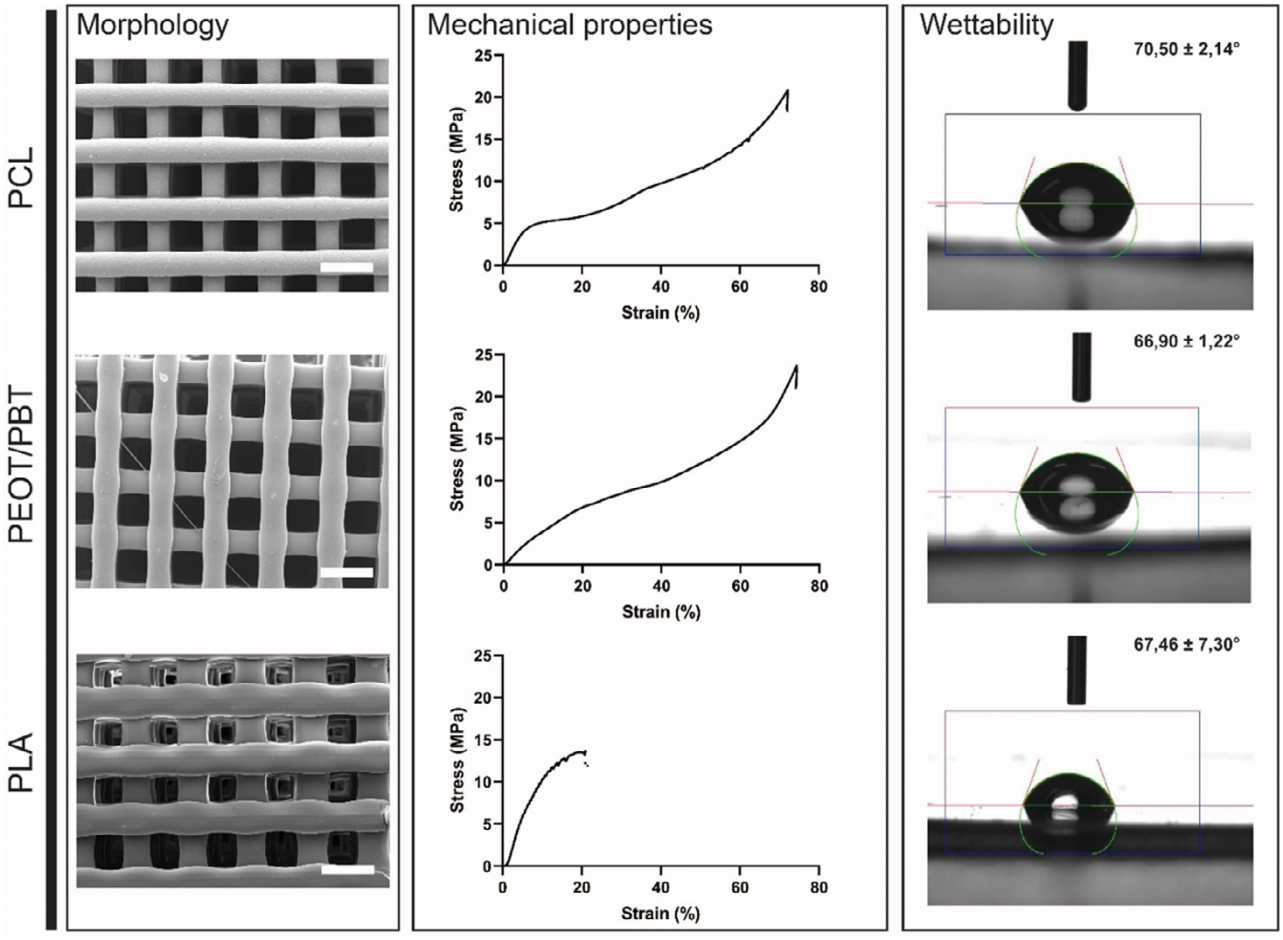
Morphological, mechanical characterization, and wettability of additive manufactured scaffolds by SEM (left panel), compressive stress–strain plots (central panel), and contact angle (right panel) with reported contact angle measurement (Scale bar is 500 μm).

The fabrication resulted in fibers of 243 ± 3 μm, 260 ± 10 μm, and 257.7 ± 14.5 μm for PCL, PEOT/PBT, and PLA, respectively (**Figure** **1**, left panel). For the mentioned materials, printing errors of 2.7 ± 1.4%, 3.7 ± 3%, and 6 ± 1% proved the high accuracy of the fabrication process. For bone tissue engineering, pore sizes bigger than 300 μm are suggested to enhance new bone and capillary formation.^[^ ^23^ ^]^ The scaffolds presented sufficient porosity with pores of 300 μm or higher. Furthermore, polymers were printed at high temperatures of 95°, 195°, and 180° for PCL, PLA, and PEOT/PBT, respectively, which might lead to degradation of the materials and consequently to a decrease of their molecular weight. Therefore, polymers' molecular weight was investigated before and after additive manufacturing through GPC (Figure S1, Supporting Information). The decrease on the molecular weight was more pronounced in PLA (from 108.489 g mol^−1^ to 34.096 g mol^−1^) followed by PEOT/PBT (55.188 g mol^−1^ to 33.085 g mol^−1^) and PCL (55.072 g mol^−1^ to 52.207 g mol^−1^). PLA is commonly blended with a secondary polymer due to the difficulty in retaining its physicochemical properties during additive manufacturing. In a previous work, the mass loss of extruded filaments of PLA was found to be 0.02% at 195° and its degradation has been attributed to hydrolysis.^[^ ^24^ ^]^ PEOT/PBT degrades according to the PEG content and initial molecular weight, which regulates the water uptake.^[^ ^25^ ^]^ In contrast, the rate of degradation of PCL is slower, which does not make it the best candidate for bone or cartilage applications.^[^ ^26^ ^]^

Water contact angle measurements of polymer‐melted films obtained by hot‐press were obtained with a goniometer system (Figure 1, right panel). Polymer pellets were melted at the deposited temperature to better resemble additive manufacturing conditions and the material state. The three materials resulted to be hydrophilic. PEOT/PBT showed a WCA of 66.9 ± 1.22° being the more hydrophilic followed by PLA with a WCA of 67.46 ± 7.3°. Lastly, PCL resulted to have the highest WCA of 70.5 ± 2.14°. Having WCA in the range of 60°–75° might be optimal to allow proteins absorption at a sufficient density without denaturation.^[^ ^27^ ^]^

To assess the mechanical properties, scaffolds were tested by compression after additive manufacturing (Figure 1, central panel). PLA resulted to be the biomaterial with the highest Young's Modulus in dry state with 84.38 ± 31.19 MPa followed by PCL and PEOT/PBT with 73.39 ± 14.98 and 42.06 ± 1.08 MPa, respectively. The maximum stress displayed by PCL was 20.84 ± 0.14 MPa, whereas 21.78 ± 1.90 MPa and 13.69 ± 0.35 MPa for PEOT/PBT and PLA, respectively. This is in line with what reported in literature.^[^ ^28^, ^29^ ^]^ For PLA, values were lower which was expected considering the brittleness of the material. Considering PLA is the material with the highest molecular weight, it is not surprising that it possesses the highest Young's Modulus closer to the one of bone (1–22.3 GPa).^[^ ^30^ ^]^ However, it was shown that the material degrades during fabrication and becomes extremely brittle.^[^ ^31^, ^32^ ^]^ During compression testing, after an initial increase in the stress, the material started to break, causing fluctuations in the stress‐strain curve on the long term. The Young's Modulus resulted to be higher than expected probably due to the deposition parameters.^[^ ^33^ ^]^ In fact, low manufacturing temperatures and low rotation speed, in combination with high molecular weight of the polymer, affect crystallization and consequently the final elastic modulus of the scaffold. Even if no statistical significance was observed between the fiber diameters of the three biomaterials, PLA showed to have a wider range of diameters, which could have an influence on the mechanical properties.

Finally, PEOT/PBT was the softest material tested with a Young Modulus that results to be the closest to the one of human articular cartilage (0.3–0.8 MPa).^[^ ^34^ ^]^ PEOT/PBT mechanical properties can be varied by altering the PEOT/PBT ratio to match the tissues characteristics.^[^ ^35^ ^]^

### 3.2 Chondrogenic and Osteogenic Differentiation Potential Analysed by Biochemical Assays

In order to evaluate the chondrogenic and osteogenic differentiation potential of hBMSCs cultured in PCL, PLA, and PEOT/PBT scaffolds, GAG assay and Safranin‐O, and ALP and alizarin red were used, respectively.

Before the biochemical assays, samples were imaged by SEM (Figure S2, Supporting Information). All the three materials in maintenance, chondrogenic and osteogenic media were covered with ECM on the interior space along the scaffold cross‐section. The matrix filled the pores and covered the filaments of the scaffolds. Overall, a denser ECM was present in the scaffolds in osteogenic medium while in chondrogenic and maintenance media the ECM looked more fibrillar.

#### 3.2.1 Chondrogenic Differentiation

**Figure 2:**
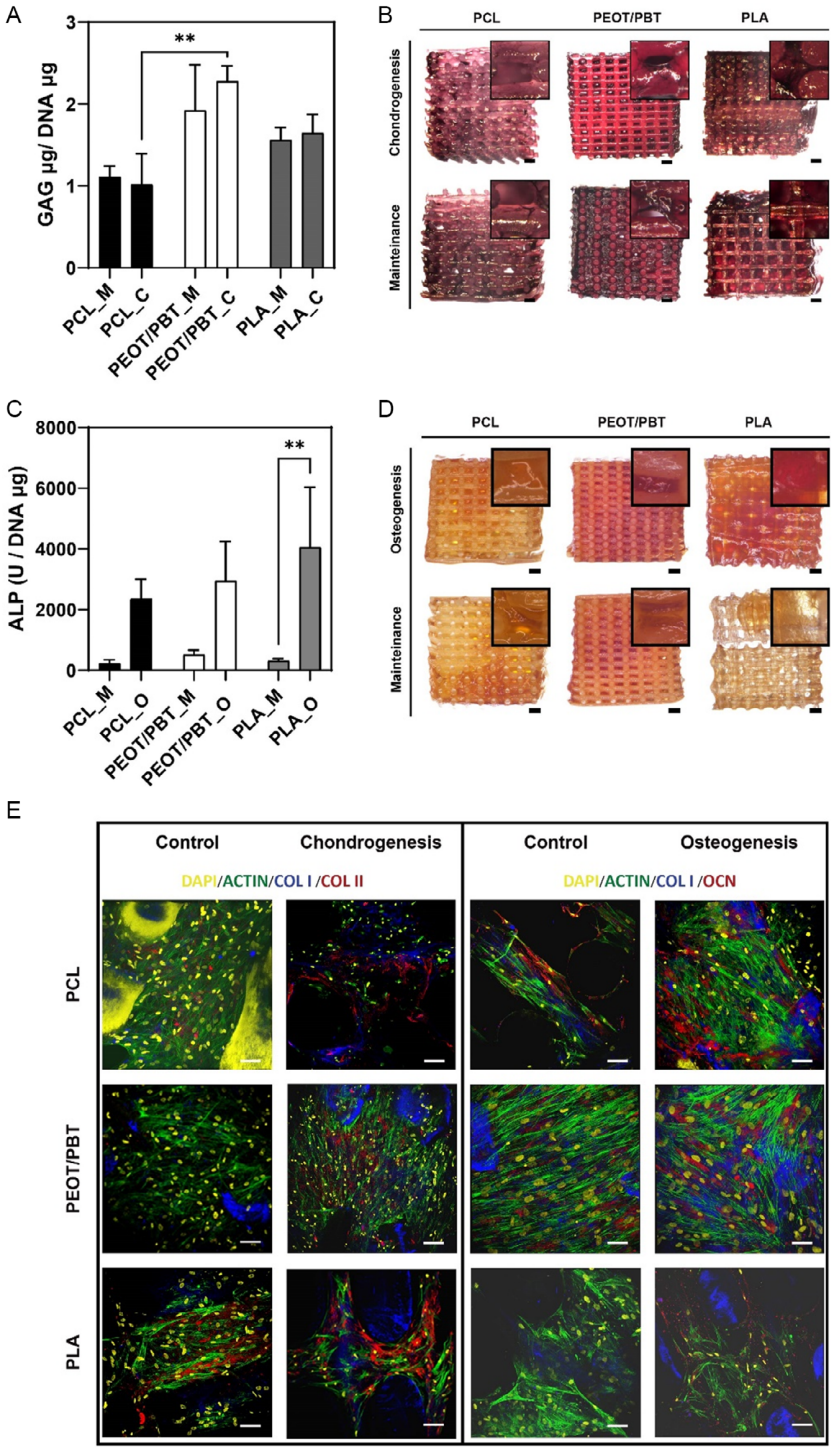
Biochemical assays. A) Normalized deposition of GAGs and B) Safranin‐O staining in PCL, PEOT/PBT, and PLA scaffolds in maintenance (_M) and chondrogenic media (_C) after 35 days of culture. C) ALP, D) Alizarin Red, and E) immunofluorescence images of cells cultured on PCL, PEOT/PBT and PLA scaffolds at day 35 in control, chondrogenic, osteogenic media. In the immunofluorescence, the cell nucleus is represented in yellow, actin in green, collagen I in blue and collagen II in red (C, D, scale bar is 500 μm; E, scale bar is 100 μm). Data is shown as mean ± SD, *n* = 3.

Proteoglycans are crucial for the correct functioning of articular cartilage as they are the main components of the ECM microenvironment.^[^ ^36^ ^]^ The GAG amount on the scaffold in the three different materials in chondrogenic and maintenance media was quantified at day 35 through a DMMB assay (**Figure** **2**A). The deposition of GAG was between 2.27 ± 0.18 μg GAG/μg DNA and 1.1 ± 0.13 μg GAG/μg DNA.

Higher GAG deposition was present in chondrogenic medium for PEOT/PBT and PLA, while PCL was the only material to show a similar deposition in the two media. This difference was more prevalent when considering total GAG content per scaffold. PEOT/PBT resulted to be the material with the highest GAG deposition (2.27 ± 0.18 μg GAG/μg DNA) in chondrogenic medium followed by PLA and PCL with a GAG deposition of 1.64 ± 0.22 μg GAG/μg DNA and 1.01 ± 0.37 μg GAG/μg DNA, respectively. A similar trend was observed in maintenance medium where PEOT/PBT resulted to be the highest with 1.92 ± 0.55 μg GAG/μg DNA followed by PLA (1.56 ± 0.15 μg GAG/μg DNA) and PCL, which resulted to be the lowest with 1.1 ± 0.13 μg GAG/μg DNA. This is in line with what observed in literature where PEOT/PBT printed scaffolds exhibit an increase in GAG concentration overtime when cultured with hBMSCs in maintenance and chondrogenic media.^[^ ^8^ ^]^ The same phenomenon was observed in PLA printed scaffolds: when seeded with chondrocytes, they showed a moderate production of GAG after 21 days.^[^ ^37^ ^]^ Previous studies^[^ ^38^ ^]^ have also reported that 3D‐printed PCL scaffolds exhibit no significant increase in GAG deposition from day 14 to day 28, a phenomenon correlated with reduced SOX9 expression.

The deposition of GAGs was further confirmed by optical observation of scaffolds stained with Safranin‐O (Figure 2B). Similarly to what was observed in the GAG assay, few differences were noted between scaffolds in maintenance and chondrogenic media. Overall, all samples showed the presence of GAGs, that is represented by a dark pink color. In particular, PEOT/PBT and PLA showed a stronger dark color compared to PCL, highlighting a GAG content also present in the scaffolds’ pores, filled with more ECM synthetized by the cells. This reflects the results of the GAG assay and might be associated with the low attachment properties of PCL observed in literature^[^ ^39^ ^]^ and associated to the materials hydrophobicity. However, the number of cells at day 35 (Figure S3, Supporting Information) showed no statistical difference between the three biomaterials in maintenance medium. We hypothesized that other cell‐material interactions are regulating and limiting the chondrogenic maturation of hBMSC‐seeded constructs. Ultimately, this remarks the need to look at a wider pool of markers to understand how to improve cell differentiation on PCL additive manufactured scaffolds.

#### 3.2.2 Osteogenic Differentiation

Alkaline phosphate (ALP) is considered an early osteogenic marker that plays an important role in hard tissue formation.^[^ ^40^ ^]^ ALP abundance was investigated at day 7 in PCL, PEOT/PBT and PLA in maintenance and osteogenic media (Figure 2C).

ALP values were higher in osteogenic medium compared to maintenance medium for the three materials. Overall, PLA and PEOT/PBT displayed higher activity in osteogenic medium being 4055.03 ± 1055.4 U μg^−1^ DNA and 2952.32 ± 1615.1 U μg^−1^ DNA, respectively, followed by PCL with 2354.42 ± 527.7 U μg^−1^ DNA. In maintenance media, the ALP concentration was lower as expected. The highest concentration was found in PEOT/PBT with 519.06 ± 119.36 U μg^−1^ DNA followed by PLA and PCL with 319.75 ± 52.58 U μg^−1^ DNA and 231.43 ± 97.11 U μg^−1^ DNA, respectively. ALP was higher and statistically significant in PLA in osteogenic medium compared to maintenance medium. This is an early indication of the osteogenic potential of PLA, which has been previously addressed in literature^[^ ^41^ ^]^ where even in absence of any surface modification the scaffolds were able to induce hBMSCs osteogenesis.^[^ ^42^ ^]^

In addition, alizarin red staining (ARS) was used to evaluate calcium rich deposits by cells at day 35 in osteogenic and maintenance media (Figure 2D). Noticeable differences were observed in PLA where in osteogenic medium the staining showed a more accentuated color than in maintenance medium. For PEOT/PBT, the material did not show any difference between maintenance and osteogenic media, most likely also due to staining of the scaffold itself, which was observed in previous works.^[^ ^43^ ^]^ Finally, PCL scaffolds showed a yellow/orange color reflecting the lack of substantial calcium deposits, which has been previously reported in literature in PCL scaffolds without additional coatings at day 21.^[^ ^44^ ^]^ Considering previous DNA and GAG results, it is hypothesized that other factors rather than attachment and proliferation are responsible for the low GAG and calcium production in this material. However, the lack of inherent osteogenic potential of PCL has been previously linked to its surface properties and low bioactivity.^[^ ^45^ ^]^

#### 3.2.3 Staining of Specialized Matrix Deposition

Bone and cartilage are characterized by the composition of specific proteins that distinguish the tissues. In articular cartilage, collagen II is a predominant component of the ECM and builds the architecture of the tissue.^[^ ^46^ ^]^ In bone, osteoblasts secrete osteocalcin and synthetize collagen I, the most abundant protein in the ECM.^[^ ^47^ ^]^ Scaffolds at day 35 were stained for collagen I and collagen II in chondrogenic medium and for osteocalcin and collagen I in osteogenic medium (Figure 2E).

In chondrogenic medium, all three materials showed higher deposition of collagen I and collagen II than in maintenance medium. PLA was the only material to show expression of collagen II also in maintenance media. In addition, PEOT/PBT and PLA displayed an extended deposition of collagen II. This result is in line with the GAG assay, as usually an high accumulation of GAG occurs simultaneously with a deposition of collagen, which is one of the building block for the growth of articular cartilage.^[^ ^48^ ^]^ This might also be due to the fact that the pores of these two materials appeared to be more filled with cells as shown in Figure S2 (Supporting Information), and therefore ECM, while PCL supported a less populated scaffold.

In osteogenic medium, the three materials showed high osteocalcin and collagen I deposition compared to maintenance medium. Especially, PCL and PEOT/PBT presented also few accumulation of these two factors in maintenance medium.

### 3.3 Chondrogenic and Osteogenic Differentiation Potential Analysed by Proteomics

To understand the biological pathways modulated by the different scaffolds upon the exposure to the different cell media, LC‐MS‐based label free proteomics experiments were performed.

Cells cultured in chondrogenic medium had a lower total protein content compared to the other media, independently of the scaffold type (Figure S4A, Supporting Information). However, the number and the variety of identified proteins was higher in the chondrogenic medium compared to other media showing probably a more efficient cell differentiation (Figure S4B, Supporting Information). No clear differences between biomaterials were observed in the total protein content, number of identified proteins and protein ID's in maintenance medium (illustrated by Figure S4 and S5, Supporting Information).

**Figure 3:**
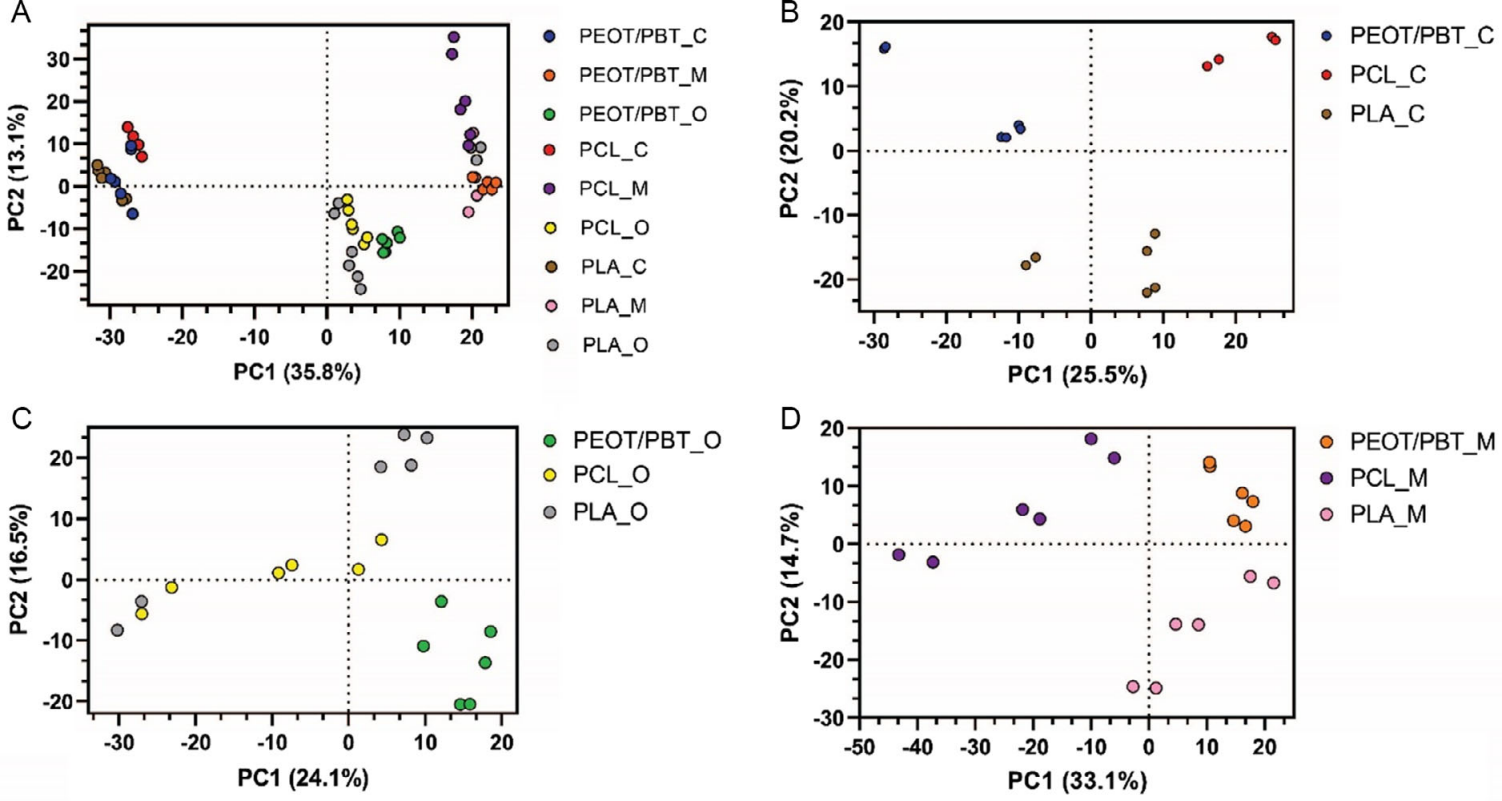
PCA Analysis of all scaffolds (PCL, PEOT/PBT, and PLA) in A) the three different media, B) in chondrogenic medium, C) in osteogenic medium, and D) in maintenance medium.

However, differential protein expression analysis revealed differences in the abundance of specific proteins between different media and biomaterials. Principal component analysis was performed to study differences in biomaterials and media at a protein level. The most pronounced variations were observed between the different types of media (**Figure** **3**A). When comparing biomaterials within each media, distinct proteomic profiles were evident for each material, besides PLA and PCL in osteogenic medium. PEOT/PBT in both maintenance and osteogenic media clustered well compared to other biomaterials (Figure 3C,D), suggesting a more specific protein profile. In chondrogenic medium (Figure 3B), all biomaterials seemed to cluster separately, with the best separation (i.e., most pronounced difference in proteomic profile) between PEOT/PBT and PCL (PC1). PLA also stood out from the other biomaterials in the second component (Figure 3B).

The PCA analyses showed that the different biomaterials had a distinct effect on stem cell differentiation. To further study the influence of the biomaterials on stem cell differentiation, differential protein expression analysis has been performed. Protein ratios were then calculated between the different conditions and ANOVA was used for hypothesis testing. Then, proteins abundancies of all proteins were plotted into a heatmap (Figure S5, Supporting Information). Clustering of samples based on cultured media was observed, showing that the proteome of samples in the same media is more alike than the proteome of the same materials in different media, which corroborates the results of the PCA plots. In addition, clustering of PLA and PEOT/PBT displayed a more similar proteome than PCL, as also observed in the PCA plots. Subsequently, significantly differentially abundant proteins (Table S1 and S2, Supporting Information) were exported to String for pathway analysis (Gene Ontology). In the figures, GO‐terms related to the most relevant biological process and molecular function pathways to chondrogenesis, osteogenesis and ECM production have been included. Full list of the pathways, including the cellular components, is present in the Supplementary information (Table S3, Supporting Information).

#### 3.3.1 Chondrogenic Medium

**Figure 4:**
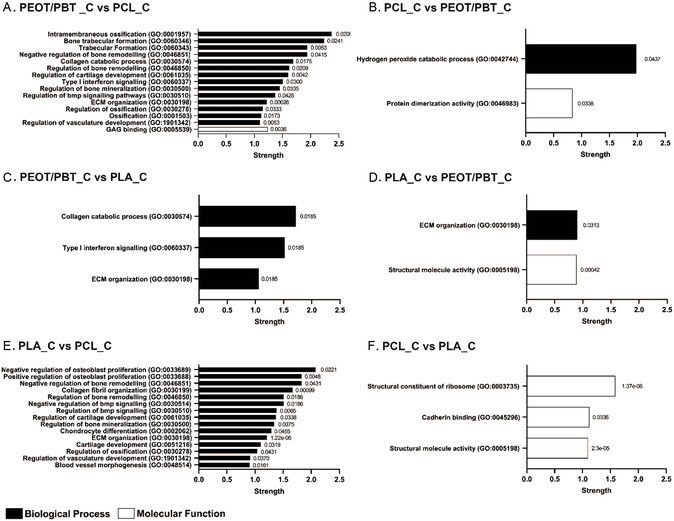
String protein pathways (most relevant GO biological process and molecular function) from upregulated proteins between materials in chondrogenic medium. A) Comparisons PEOT/PBT_C versus PCL_C, B) PCL_C versus PEOT/PBT_C, C) PEOT/PBT_C versus PLA_C, D) PLA_C versus PEOT/PBT_C, E) PLA_C versus PCL_C, and F) PCL_C versus PLA_C with columns summarizing the selected pathways with their GO term, strength, and false discovery rate at the end of the respective columns.

Looking at the comparisons between materials in chondrogenic medium, it was possible to determine the upregulated proteins and their related pathways involved in chondrogenesis (**Figure** **4**, Figure S6, Supporting Information). It was observed that PEOT/PBT, a widely used material in cartilage applications^[^ ^49^, ^50^, ^51^ ^]^ with Young's modulus similar to cartilage, showed upregulation of various cartilage related pathways when compared to PCL (Figure 4A). PEOT/PBT induced regulation of cartilage development (GO:0061035), ECM organization (GO:0030198), as well as glycosaminoglycan binding pathways (GO:0005539). Among the upregulated proteins, thrombospondin 2 (THBS2), GremLin 1 (GREM1), matrix metalloproteinase 2 (MMP2) and hyaluronan and proteoglycan link protein 1 (HAPLN1) have been previously studied for their role in the differentiation of hBMSC into chondrocytes and in cartilage development.^[^ ^52^, ^53^, ^54^, ^55^, ^56^ ^]^ This is in line with our previous GAG measurements that show more GAG deposition in PEOT/PBT compared to PCL in chondrogenic medium. Interestingly, PEOT/PBT induced also pathways related to intramembranous ossification (GO:0001957), bone trabecular formation (GO:0060346), regulation of bone remodeling (GO:0046850), regulation of BMP signaling pathway (GO:0030510), regulation of bone mineralization (GO:0030500) and ossification (GO:0001503). This might indicate that at the end of the differentiation hBMSCs assume a hypertrophic phenotype on PEOT/PBT scaffolds, also proved by the upregulation of matrix metalloproteinase (MMP‐13) and cysteine‐rich protein 61 (CYR61), a marker for terminally differentiated chondrocytes.^[^ ^57^, ^58^ ^]^ Together with MMP‐13, cathepsin D (CSTD) and cathepsin K (CTSK) are involved in matrix degradation at the osteochondral junction area and were found limited to hypertrophic chondrocytes.^[^ ^59^, ^60^ ^]^

On the contrary (Figure 4B), PCL upregulated proteins related to the hydrogen peroxide catabolic process pathway (GO:0042744) and associated as a molecular function to the protein dimerization activity pathway (GO:0046983), which are not significant to the chondrogenic process. When compared to PLA (Figure 4C), PEOT/PBT induced collagen catabolic processes (GO:0030574), type I interferon signaling (GO:0060337) and extracellular organization (GO:0030198), showing also in this comparison the upregulation of GREM1, MMP2, and HAPLN1 alongside MMP13. The interferon signaling pathway was also upregulated, which is a pathway related to the immunomodulatory activity of hBMSCs. PLA induced pathways (Figure 4D) related to extracellular matrix organization (GO:0030198) and structural molecule activity (GO:0005198), which showed the presence of many proteins related to cell adhesion like vitronectin (VTN), dermatopontin (DPT), prolargin (PRELP), and transforming growth factor beta 1 (TGFB1). While vitronectin is known to promote osteogenic differentiation,^[^ ^61^ ^]^ DPT, PRELP, and TGFB1 are associated to cartilage. DPT is reported in chondrocyte culture^[^ ^62^ ^]^ where its function might be connected to collagen II in maintaining the mechanical strength or elasticity of the cartilage and that is why it is also a component of the collagen‐containing extracellular matrix. PRELP is expressed in articular cartilage^[^ ^63^ ^]^ and the TGF‐β family is known to play a role during chondrogenesis in vitro by committing hBMSCs to mesenchymal chondroprogenitor cells.^[^ ^64^ ^]^ Interestingly, within the ECM proteins, also S100A10, S100A9, and S100A7 were upregulated. The S100 protein expression was previously localized in late proliferative and pre‐hypertrophic chondrocytes of the mouse growth plate^[^ ^65^ ^]^ and has shown to suppress hypertrophic differentiation and mineralization of chondrocytes in hBMSC chondrogenic differentiation.

Finally, when comparing PLA to PCL in chondrogenic medium (Figure 4E), PLA showed a number of pathways related to chondrogenesis such as collagen fibril organization (GO:0030199), regulation of cartilage development (GO:0061035), chondrocyte differentiation (GO:0002062) and cartilage development (GO:0051216). Within these pathways, PLA showed to upregulate GREM1, cartilage oligomeric matrix protein (COMP), collagen III (COL3A1), collagen VI (COL6A1), procollagen C‐endopeptidase enhancer 1 (PCOLCE) and disintegrin and metalloproteinase with thrombospondin motifs 2 (ADAMTS2). It has been reported that COMP is involved in the matrix assembly during the chondrogenesis of hBMSCs by interacting with aggrecans and collagens,^[^ ^66^ ^]^ but it might also have a role in osteogenesis by binding to BMP‐2 and enhancing its activity.^[^ ^67^ ^]^ PCOLCE and ADAMTS2 and the collagen isoforms COL3A1 and COL6A1 are proteins involved in the support of collagen biosynthesis and extracellular matrix deposition during skeletal tissue development.^[^ ^68^, ^69^ ^]^ Similarly to when PEOT/PBT was compared to PCL, also in this comparison proteins associated to bone pathways, such as regulation of osteoblast differentiation (GO:0033689), regulation of bone remodeling (GO:0046850), regulation of bone mineralization (GO:0030500), and regulation of ossification (GO:0030278) were present and proteins associated to cartilage such as CYR61 and TGFB1 were found. When comparing PCL with PLA (Figure 4F), PCL showed instead pathways connected to the structural constituent of ribosome (GO:0003735), cadherin binding (GO:0045296), and structural molecule activity (GO:0005198). PCL induced upregulation of two calcium‐binding protein, S100A11 and annexin 4 (ANXA4), which have a role in early osteogenesis and chondrogenesis^[^ ^70^ ^]^ as well as a large group of ribosomal proteins (RPS15, RPS17, RPL39, RPL27, RPL35, RPL34, and RPL36).

Considering that the PCA analysis showed the chondrogenic medium to be the condition where the materials had the most pronounced differences in proteomic profiles, it was expected to observe the regulation of many different pathways. Overall, the String analysis on the upregulated proteins in chondrogenic medium seems to indicate that PEOT/PBT and PLA are more suitable candidates for chondrogenic applications compared to PCL. When PCL was compared to PEOT/PBT and PLA, no pathways related to cartilage were found. However, it was noted the upregulation of different proteins related to early chondrogenesis such as annexin a4 (ANXA4)^[^ ^71^ ^]^ and MAC‐inhibitory protein (CD59).^[^ ^72^ ^]^ This might indicate that further functionalization of the material such as growth factors or peptides addition could be beneficial to obtain a more mature phenotype and more ECM production. In contrast, when PEOT/PBT and PLA were compared to PCL, various pathways related to cartilage were present, showing the upregulation of numerous proteins involved in the chondrogenic differentiation of hBMSCs such as GREM1, MMP2, and HAPLN1 for PEOT/PBT_C and GREM1, COMP, and PCOLCE for PLA_C, respectively. In both comparisons (PEOT/PBT_C vs. PCL_C and PLA_C vs. PCL_C), there were also pathways related to bone. This is likely due to the fact that proteins involved in the chondrogenic differentiation can play a role in the overall skeletal tissue development. However, when PEOT/PBT_C was compared with PLA_C, MMP13, CYR61, CTSD, and CTSK were upregulated, indicating a hypertrophic chondrocytes phenotype. This suggests that PEOT/PBT could be used to recapitulate endochondral ossification or as an interface in the osteochondral junction.

#### 3.3.2 Osteogenic Medium

**Figure 5:**
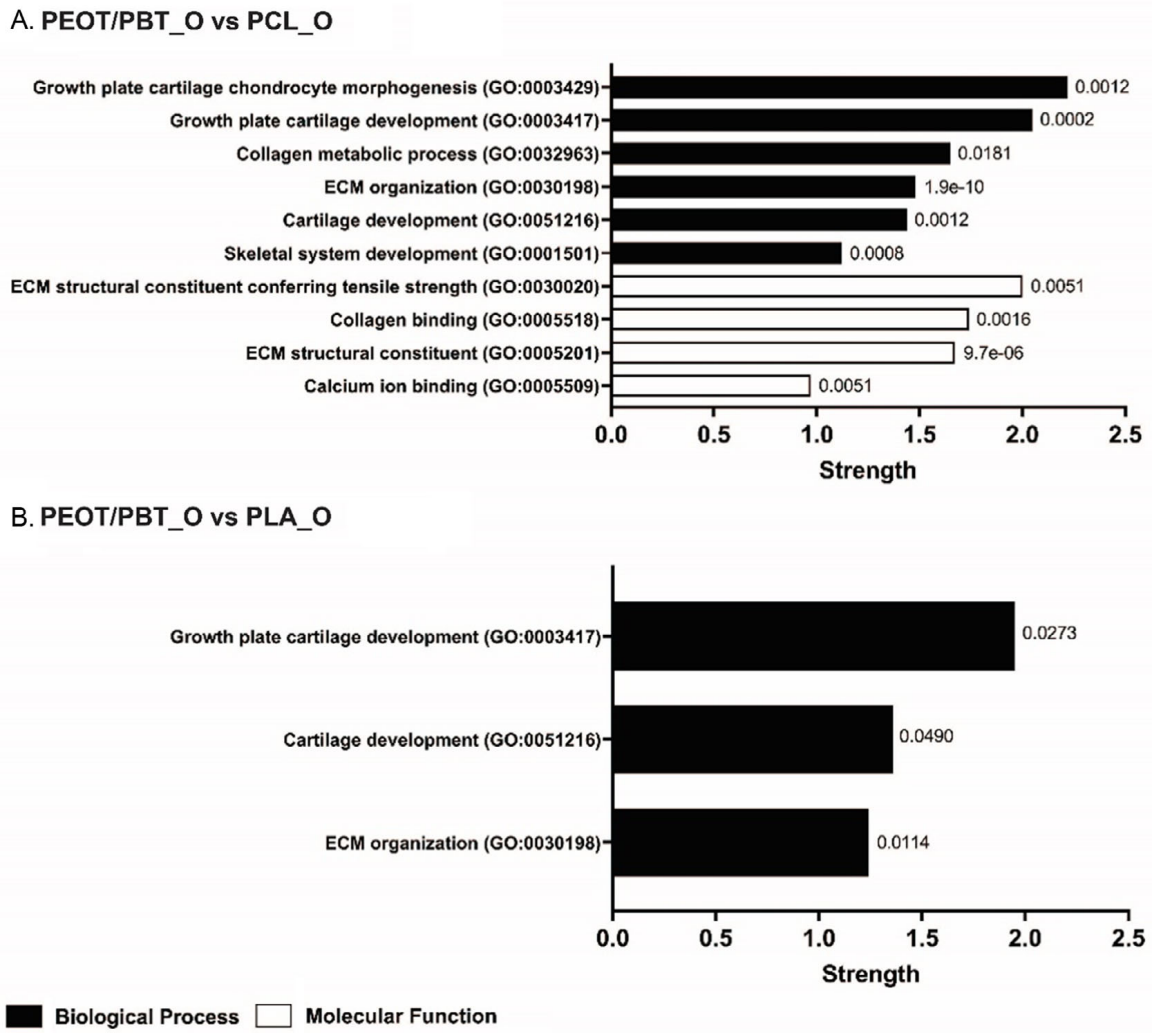
String protein pathways (most relevant GO biological process and molecular function) from upregulated proteins between materials in osteogenic medium. Comparisons A) PEOT/PBT_O versus PCL_O and B) PEOT/PBT_O versus PLA_O with columns summarizing the selected pathways with their GO term, strength, and false discovery rate at the end of the respective columns.

When analyzing the results of the osteogenic differentiation through ALP and alizarin red, it was not clear if a material was outperforming the others. While PLA had a higher ALP, which is an early marker of osteogenesis, PEOT/PBT showed also a strong alizarin red stain at a later stage and like PCL, OCN deposition in osteogenic medium. Hence, with the aid of proteomics, it was possible to analyze more in deep the differentiation processes in the scaffolds (**Figure** **5**, Figure S7, Supporting Information).

When cultured in chondrogenic medium and compared to PCL under the same conditions, PEOT/PBT induced various chondrogenesis‐related proteins. Likewise, when compared to PCL in osteogenic medium (Figure 5A), PEOT/PBT induced growth plate cartilage chondrocyte morphogenesis (GO:0003429), growth plate cartilage development (GO:0003417), collagen metabolic process (GO:0032963), ECM organization (GO:0030198), cartilage development (GO:0051216), skeletal system development (GO:0001501) as well as molecular function pathways such as ECM structural constituent conferring tensile strength (GO:0030020), collagen binding (GO:0005518), ECM structural constituent (GO:0005201) and calcium ion binding pathways (GO:0005509). Among these pathways, COL6A1, COL6A2, COL6A3, HAPLN1 and MMP13 proteins were upregulated and associated to chondrogenesis.^[^ ^56^, ^73^ ^]^ Metalloproteinase inhibitor 1 (TIMP‐1) was also observed, a protein involved in the downregulation of Sox9 and inhibition of osteogenic differentiation of hBMSC^[^ ^74^ ^]^ as well as endosialin (CD248), a negative regulator of bone formation in mice.^[^ ^75^ ^]^ However, VCAN, ectonucleotide pyrophosphatase/phosphodiesterase family member 1 (ENPP1) and latent‐transforming growth factor beta‐binding protein 2 (LTBP2) proteins, which are associated with early osteogenesis,^[^ ^76^, ^77^ ^]^ were also upregulated. On the contrary, PCL‐induced upregulated proteins associated only with the cellular component of the ECM exosome, ECM space and vesicles (Figure S7B).

When compared to PLA in osteogenic medium (Figure 5B), PEOT/PBT upregulated proteins were connected to growth cartilage plate development (GO:0003417), cartilage development (GO:0051216) and ECM organization (GO:0030198). Also in this case, PEOT/PBT showed various pathway associated to chondrogenesis and the presence of upregulated proteins COL6A1, COL6A3, MMP13. TIMP‐1, and ENPP1 was also shown. When analyzing the upregulated proteins in PLA compared to PEOT/PBT (Figure S7D, Supporting Information), it was observed that these materials upregulated proteins related to the cellular components of collagen‐containing ECM and ECM. Within these pathways, GREM1, THBS2, angiopoietin‐related protein 2 (ANGPTL2), spondin‐1 (SPON1), insulin‐like growth factor‐binding protein 10 (CYR61) were present. From the biochemical assays, PLA and PEOT/PBT showed a comparable performance in osteogenic medium, which is also reflected in the proteomics comparison between PLA and PEOT/PBT. However, in osteogenic medium, PLA showed the upregulation of many proteins also related to endothelial cell sprouting and angiogenesis (ANGPTL2, SPON1, and CYR61),^[^ ^78^, ^79^, ^80^ ^]^ which might propose it as a better candidate for bone applications compared to PEOT/PBT, which showed the upregulation of various cartilage‐related proteins even in osteogenic medium. In fact, ALP quantification showed that PLA had the highest ALP abundance compared to the other materials.

Looking at the comparison between PCL and PLA (Figure S7E, Supporting Information), no significant enrichment was detected in the pathways. This might indicate that PCL shares a similar proteomic profile with PLA during osteogenic differentiation. However, in the case of PLA (Figure S7F, Supporting Information), even if no pathways were detected, HAPLN1 and VCAN, proteins involved in the ECM structure of the early stages of osteogenesis were found upregulated.^[^ ^81^ ^]^

Similarly as for chondrogenic medium, the String software was used to analyze the pathways related to the upregulated proteins between two materials in osteogenic medium. A lack of osteogenic‐specific pathways was observed when materials were compared to each other. This might indicate that the three materials have similar expression of osteogenic‐related proteins, which was shown in the PCA analysis, or that at the end of the differentiation the cells are still at an early stage of osteogenesis, as demonstrated by the expression of ENPP1 and LTBP2 in PEOT/PBT_O versus PCL_O.

#### 3.3.3 Maintenance Medium

**Figure 6:**
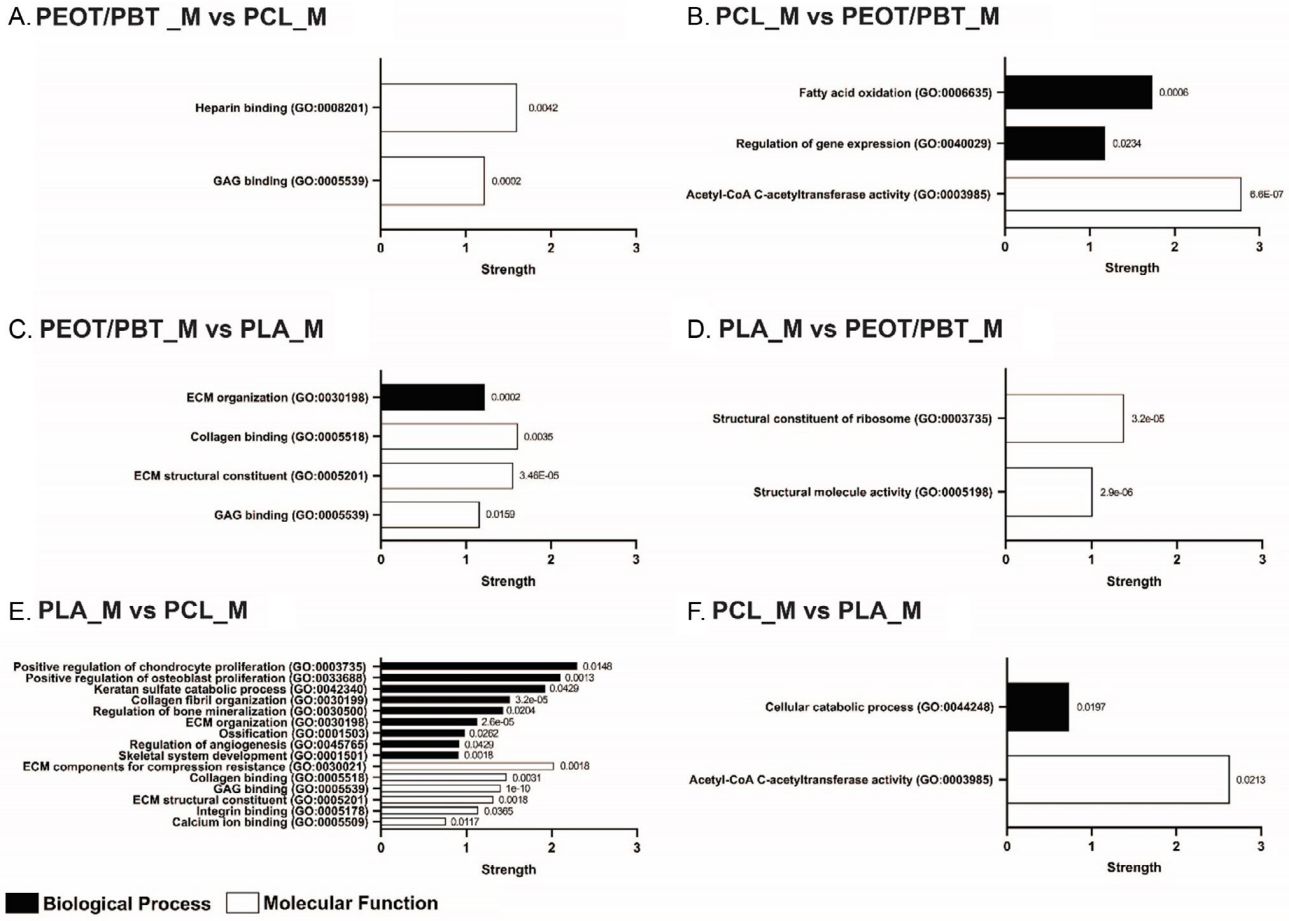
String protein pathways (most relevant GO biological process and molecular function) from upregulated proteins between materials in maintenance medium. Comparisons A) PEOT/PBT_M versus PCL_M, B) PCL_M versus PEOT/PBT_M, C) PEOT/PBT_M versus PLA_M, D) PLA_M versus PEOT/PBT_M, E) PLA_M versus PCL_M, and F) PCL_M versus PLA_M with columns summarizing the selected pathways with their GO term, strength and FDR at the end of the respective columns.

After analyzing chondrogenic and osteogenic media, the proteome of cells cultured in the different materials were compared in maintenance medium and the pathways related to the resulting upregulated proteins were analyzed (**Figure** **6**, Figure S8, Supporting Information). Considering the previous results from the differentiation media, it was hypothesized that PEOT/PBT was a good candidate for cartilage applications. In fact, when compared to PCL in maintenance medium (Figure 6A), pathways related to heparin binding (GO:0008201) and glycosaminoglycan binding (GO:0005539) as well as cellular component such as collagen‐containing extracellular matrix were found. PEOT/PBT induced the upregulation of PCOLCE, HAPLN1, THBS2, MMP2, secreted frizzled‐related protein 1 (SFRP1), CYR61 and FBLN5. While PCOLCE, HAPLN1, THBS2, MMP2, and ITIH2 have been noted for their role in skeletal tissue, SFRP1, CYR61 and FBLN5 might be involved in angiogenesis.^[^ ^82^ ^]^ When compared to PLA (Figure 6C), PEOT/PBT showed upregulation of proteins involved in ECM organization (GO:0030198), collagen binding (GO:0005518), ECM structural constituent (GO:0005201) and glycosaminoglycan binding (GO:0005539) such as COL6A1, COL6A3, VCAN, decorin (DCN), TIMP1, SPON1, PCOLCE, SFRP1, and HAPLN1. In addition to collagen VI, DCN is an important proteoglycan in the mechanical function of the pericellular matrix and acts as a biochemical modulators during chondrogenesis.^[^ ^83^ ^]^

When compared to PEOT/PBT in maintenance medium (Figure 6D), PLA upregulated proteins in the structural constituent of ribosome (GO:0003735) and in structural molecular activity (GO:0005198) such as large subunit ribosomal protein (RPL4, RPL13A, RPL18, RPL6, RPL7A, RPL18A), which are not strong indicators of a tendency of the seeded cells toward a skeletal tissue application but indicate high levels of protein translation.^[^ ^84^ ^]^ However, ADAMTS2 and CYR61 were also upregulated when PLA was compared to PCL in chondrogenic and in osteogenic medium, respectively. In addition, when compared to PCL (Figure 6E), PLA induced pathways related to cartilage such as positive regulation of chondrocyte proliferation (GO:0003735), keratin sulfate catabolic process (GO:0042340) and glycosaminoglycan binding (GO:0005539) as well as to bone like positive regulation of osteoblast proliferation (GO:0033690), regulation of bone mineralization (GO:0030500), ossification (GO:0001503) and calcium ion binding (GO:0005509). For cartilage‐related pathways, COMP, HAPLN1, VTN, PRELP, VCAN, lumican (LUM), CYR61, PCOLCE, tetranectin (CLEC3B), latent‐transforming growth factor beta‐binding protein 2 (LTBP2), lactotransferrin (LTF), SERPINC1 and APOH were upregulated, whereas for bone, ENPP1, VCAN, SCIN, FLBN5, COMP, filaggrin (FLG), LTBP2, CYR61, CLEC3B, and LTF. Proteins related to the regulation of angiogenesis were also upregulated (serpin Family F Member 1 (SERPINF1), apolipoprotein H (APOH), RAS related (RRAS), keratin 1 (KRT1), FBLN5, showing the potential of this material to modulate angiogenesis. Also in this case, components of the RPL family such as RPL7A, RPL4, RPL14, RPL18A, RPL18A, and RPL13A were present, evidencing again high protein translation.

Finally, even if we did not observe a particular tendency for PCL toward chondrogenesis or osteogenesis, it was noted that proteins associated to fatty acid beta‐oxidation (GO:0006635), regulation of gene expression (GO:0040029) and acetyl‐CoA C‐acetyltransferase activity (GO:0003985) were upregulated when compared with PEOT/PBT (Figure 6B) and pathways related to cellular catabolic process (GO:0044248) and acetyl‐CoA C‐acetyltransferase activity (GO:0003985) when compared to PLA (Figure 6F), respectively.

String analysis in maintenance medium was primarily performed to observe if without the addition of growth factors, the materials could induce chondrogenic or osteogenic differentiation. In chondrogenic and osteogenic medium, no differentiation‐related pathways were observed in PCL when compared to PEOT/PBT and PLA. Also in maintenance medium, PCL did not upregulate any pathways related to chondrogenesis or osteogenesis when compared to the other two materials. Interestingly, PEOT/PBT_M showed a pool of proteins involved in chondrogenesis such as PCOLCE, HAPLN1, THBS2, MMP2, and PCOLCE, HAPLN1 DCN and VCAN when compared to PCL_M and PLA_M, respectively, thus showing the potential to induce an early chondrogenic phenotype without the assistance of growth factors. This can be corroborated by the presence of the glycosaminoglycan binding pathways in both comparisons alongside the high GAG concentration observed in the GAG assay in maintenance medium. Similarly, PLA_M showed multiple pathways associated with chondrogenesis and osteogenesis compared to PCL_M. By the presence of proteins (SERPINF1, RRAS, KRT1, and FBLN5) associated with vascularization and angiogenesis also found in osteogenic medium, it might be hypothesized that PLA could be more suitable for bone applications where vascularization is essential for tissue development and healing. Considering also the lack of skeletal tissue‐related pathways in PLA_M versus PEOT/PBT_M, it is suggested that PEOT/PBT could more efficiently induce chondrogenesis compared to PLA.

This study focuses on determining the chondrogenic and osteogenic potential of additive manufacturing materials in vitro using proteomics and traditional biochemical assay. Although the study does not predict their response in large animals or humans, it serves as an initial assessment for future biomaterial skeletal tissue applications. In future studies, these findings will be reinforced through pre‐clinical data taking into account also the foreign body response. Emphasizing the importance of proteomics in in vitro screening, this work highlights its role in providing a comprehensive understanding of cell‐biomaterial interactions also for other applications such as using these materials for 3D advanced in vitro models.

## 4 Conclusions

We have demonstrated the successful fabrication of additive manufactured scaffolds composed by PCL, PEOT/PBT, or PLA, which showed to be all hydrophilic and to have Young's moduli in the range of bone and cartilage for PLA and PCL and PEOT/PBT, respectively. Overall, in chondrogenic medium, biochemical assays such as GAG assay and safranin‐O showed that PEOT/PBT and PLA were more effective than PCL at promoting chondrogenesis of hBMSCs. In osteogenic medium, PEOT/PBT and PLA also outperformed PCL in supporting cell osteogenic differentiation. Proteomics analysis revealed that all biomaterials showed distinct proteomics profiles, especially in chondrogenic medium. At the end of the culture, no differentiation‐related pathways were observed in PCL when compared to PEOT/PBT and PLA in chondrogenic and osteogenic media, corroborating the results of the biochemical assays where it was outperformed by the other two materials. PEOT/PBT exhibited potential in directing cells toward a chondrogenic phenotype even without soluble differentiation factors and the presence of proteins associated with hypertrophic chondrocytes suggested the use of this material as an interface for the osteochondral junction. PLA showed potential for cartilage and bone application, also demonstrating its ability to enhance vascularization and angiogenesis‐related pathways. This strongly suggests that this material holds promise for a more substantial and favorable impact in bone regeneration.

This study enhanced our understanding on how cells interact in vitro with 3D printed PCL, PEOT/PBT and PLA scaffolds and highlights how proteomics can offer a more integrated and complex view of the biological processes at the interface. Proteomics can enrich the biological characterization of 3D printed materials and help evaluate the ideal candidates for cartilage and bone tissue engineering applications as well as aid the design of new effective biomaterials for skeletal tissue applications.

## Conflict of Interest

The authors declare no conflict of interest.

## Author Contributions

C.T.: Methodology, Investigation, Writing‐original draft, Formal analysis. R.M.: Investigation, Data curation, Writing‐review and editing, Formal Analysis. S.C.‐E.: Conceptualization, Methodology, Writing‐review and editing, Supervision. B.C.‐P.: Conceptualization, Methodology, Data curation, Writing‐review and editing, Supervision. L.M.: Conceptualization, Methodology, Writing‐review and editing, Supervision, Project administration, Funding acquisition.

## Supporting information

Supplementary Material

## Data Availability

The data that support the findings of this study are available from the corresponding author upon reasonable request.
