# Supplementary material for: A Proteomic Approach to Determine Stem Cell Skeletal Differentiation Signature on Additive Manufactured Scaffolds
Source: Small Sci. 2024 Jun 2;4(7):2300316. doi: 10.1002/smsc.202300316 (PMC11935236; doi:10.1002/smsc.202300316)
Supplement: Supplementary file 1 — Supplementary Material [file SMSC-4-2300316-s001.pdf]

A proteomic approach to determine stem cell skeletal differentiation signature on additive manufactured scaffolds

Supplementary Information

Clarissa Tomasina[1], Ronny Mohren[2], Sandra Camarero-Espinosa[1,3,4], Berta Ciliero-Pastor[1,2] and Lorenzo Moroni[1].

[1]*MERLN Institute for Technology-inspired Regenerative Medicine, Complex Tissue Regeneration Department, Maastricht University, P.O. Box 616, 6200MD Maastricht, The Netherlands*
[2]*The Maastricht MultiModal Molecular Imaging Institute (M4i), Division of Imaging Mass Spectrometry, Maastricht University, The Netherlands*
[3]*POLYMAT University of the Basque Country UPV/EHU Avenida Tolosa 72, Donostia 20018, Gipuzkoa, Spain*
[4]*IKERBASQUE, Basque Foundation for Science, 48009 Bilbao, Spain*

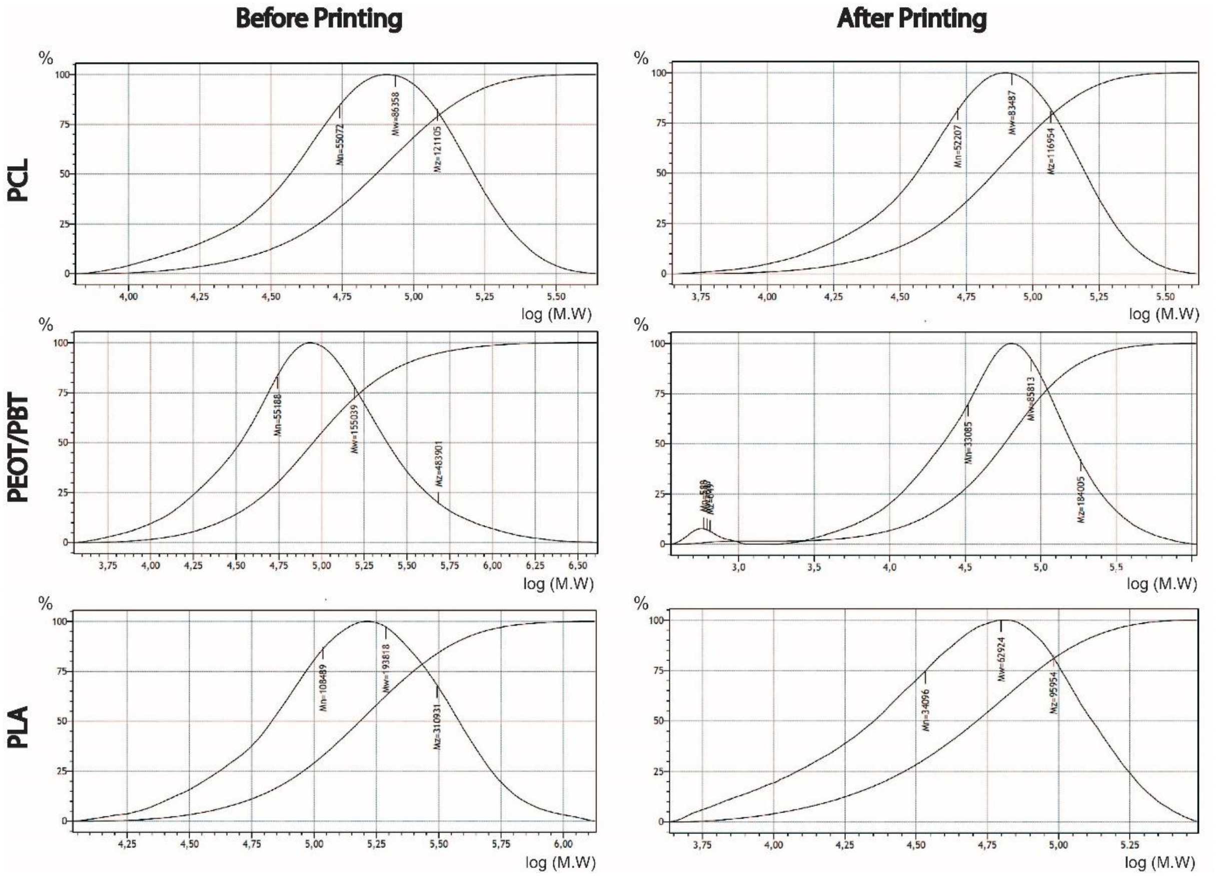

Figure S1. GPC plots (logarithm of molecular weight vs normalized intensity) of the materials before (left) and after (right) 3D printing showing a slight decrease of the molecular weight. M_w (weight-average molecular weight), M_n (polymer number-average molecular weight) and M_z (higher average molecular weight).

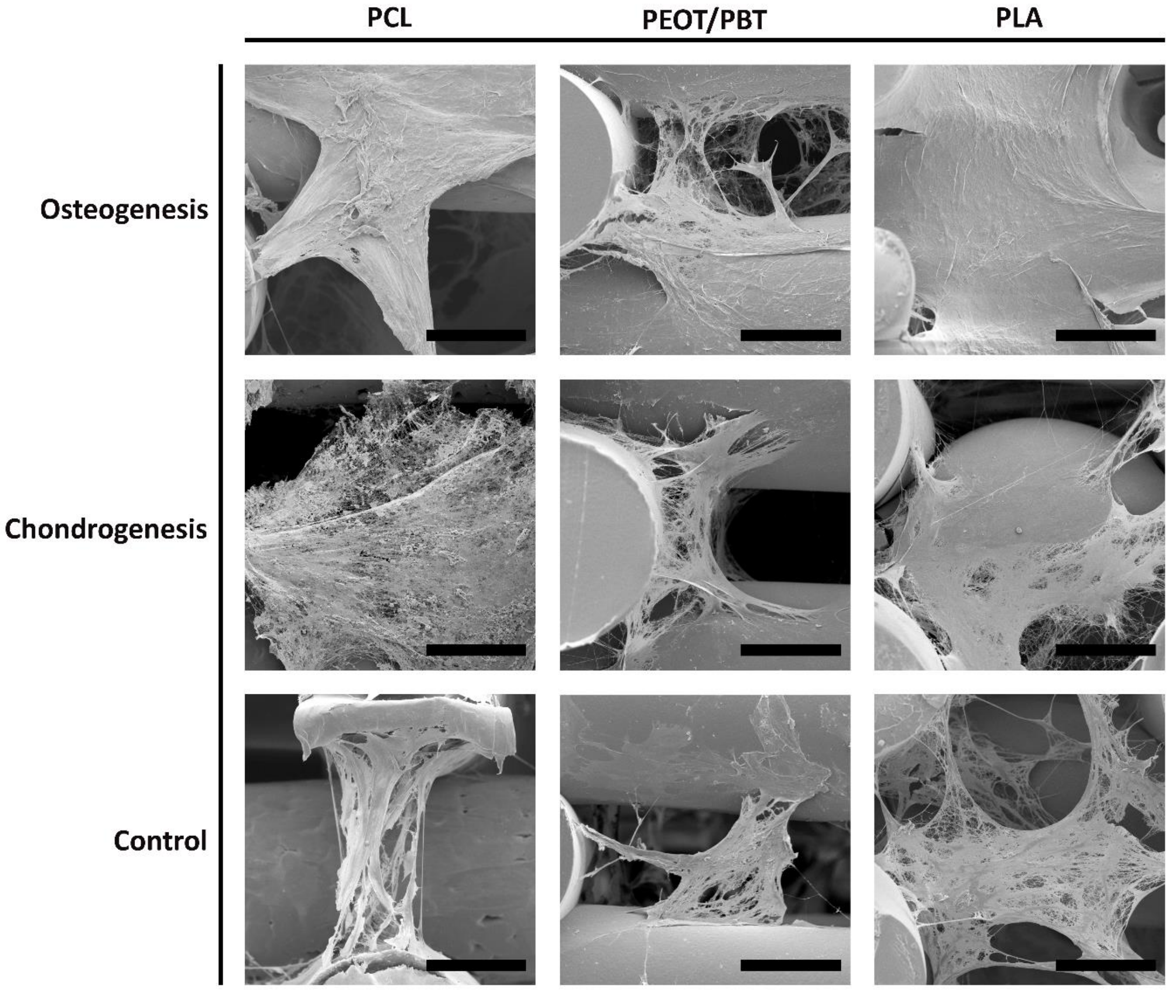

Figure S2. SEM images of hBMSCs cultured for 35 days in PCL, PEOT/PBT and PLA scaffolds in different media (Scale bar is 100 µm).

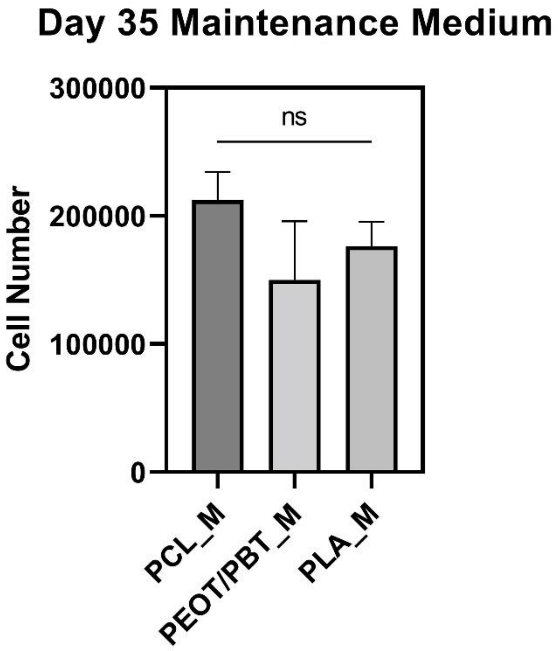

Figure S3. Cell Number in maintenance medium at day 35 (end point of culture).

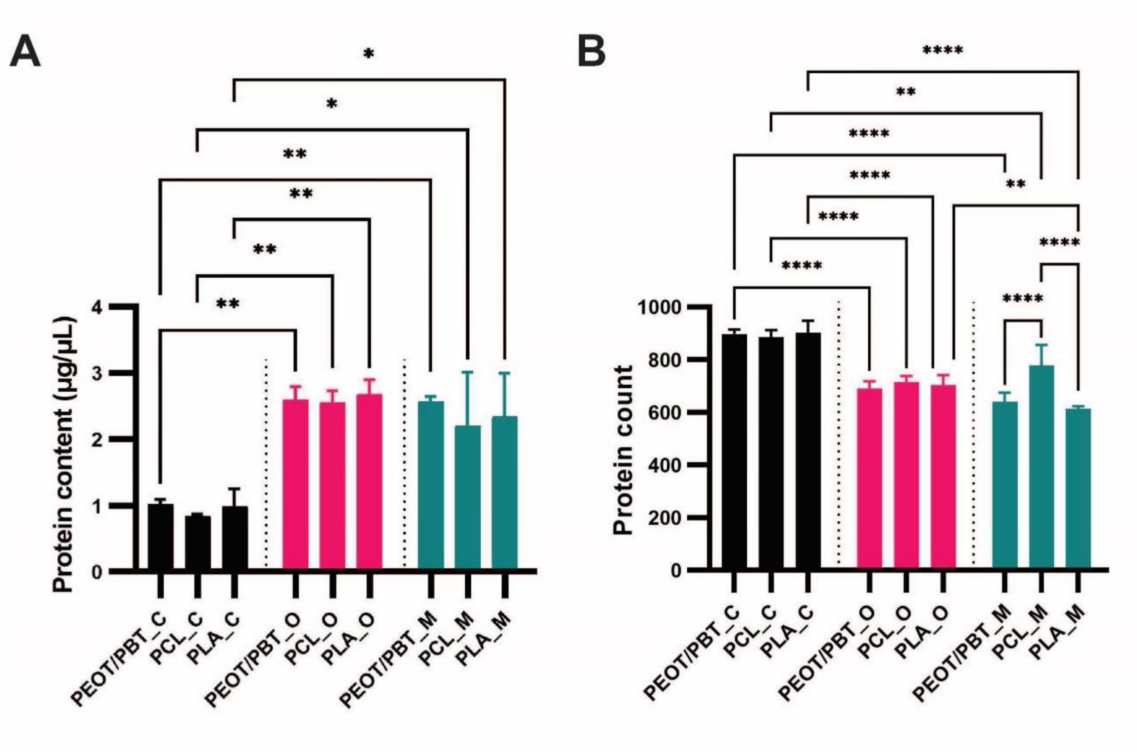

Figure S4. Total protein content quantified by Bradford assay (A) and number of proteins identified (B) by LC/MS.

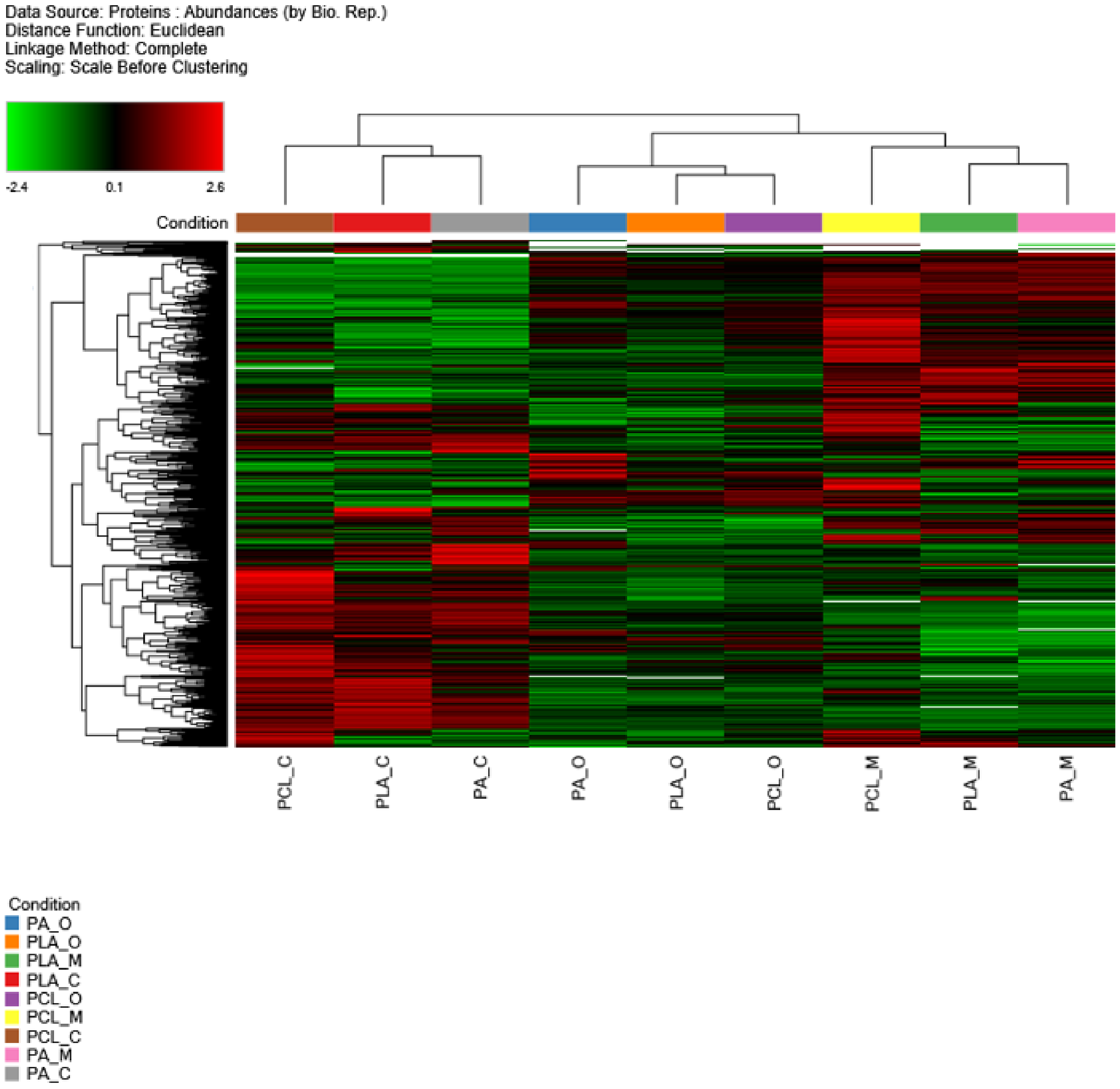

Figure S5. Heat map of all proteins in PCL, PEOT/PBT (PA) and PLA in chondrogenic, osteogenic and maintenance medium with replicates combined.

All_Proteins_with_quan_data.pdf

Table S1. Abundance and abundance ratios of all proteins (upregulated and down regulated).

Protein lists.xlsx

Table S2. List of significant differentially proteins in the comparisons between materials used for String Analysis.

List all pathways.xlsx

Table S3. List of the GO-terms (biological process, molecular function and cellular component) of the resulting pathways from the String analysis software in all comparisons in chondrogenic, osteogenic and maintenance medium.

A. PEOT/PBT_C vs PCL_C

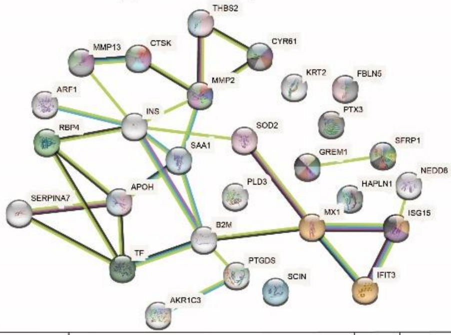

GO-Term	Description	Color	Strength	FDR
GO:0001957	Intramembraneous ossification	(blue)	2.37	0.0209
GO:0060346	Bone trabecular formation	(red)	2.24	0.0241
GO:0060343	Trabecula formation	(green)	1.94	0.0053
GO:0046851	Negative regulation of bone remodeling	(yellow)	1.94	0.0415
GO:0030574	Collagen catabolic process	(magenta)	1.69	0.0175
GO:0046850	Regulation of bone remodeling	(dark green)	1.62	0.0209
GO:0061035	Regulation of cartilage development	(light blue)	1.6	0.0042
GO:0060337	Type I interferon signaling	(gold)	1.5	0.0300
GO:0030500	Regulation of bone mineralization	(purple)	1.45	0.0335
GO:0030510	Regulation of bmp signaling pathway	(dark red)	1.37	0.0425
GO:0030198	Extracellular matrix organization	(grey)	1.22	0.00026
GO:0030278	Regulation of ossification	(black)	1.15	0.0333
GO:0001503	Ossification	(light green)	1.12	0.0173
GO:1901342	Regulation of vasculature development	(pink)	1.1	0.0053
GO:0005539	Glycosaminoglycan binding	(light grey)	1.24	0.0036

B. PCL_C vs PEOT/PBT_C

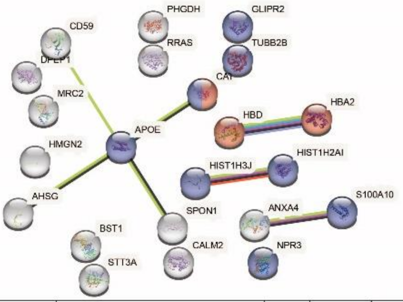

GO-Term	Description	Color	Strength	FDR
GO:0042744	Hydrogen peroxide catabolic process	(blue)	1.98	0.0437
GO:0046983	Protein dimerization activity	(red)	0.84	0.0338

D. PLA_C vs PEOT/PBT_C

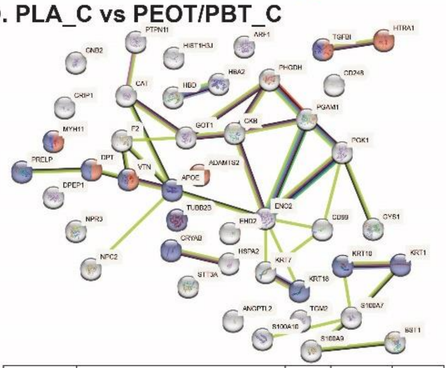

GO-Term	Description	Color	Strength	FDR
GO:0030198	Extracellular matrix organization	(blue)	0.9	0.0313
GO:0005198	Structural molecule activity	(red)	0.89	0.00042

C. PEOT/PBT_C vs PLA_C

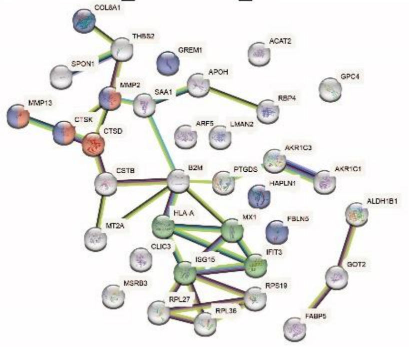

GO-Term	Description	Color	Strength	FDR
GO:0030574	Collagen catabolic process	(blue)	1.72	0.0185
GO:0060337	Type I interferon signaling pathway	(red)	1.52	0.0185
GO:0030198	Extracellular matrix organization	(green)	1.06	0.0185

E. PLA_C vs PCL_C

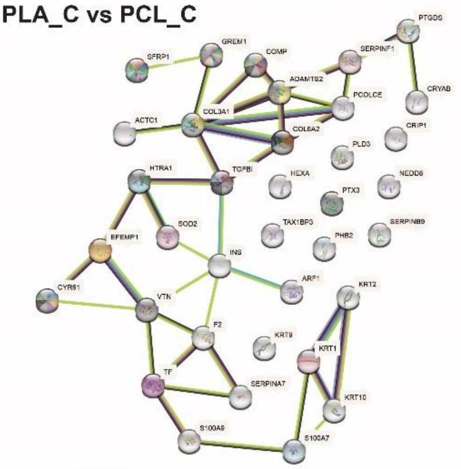

GO-Term	Description	Color	Strength	FDR
GO:0033689	Negative regulation of osteoblast proliferation	(blue)	2.08	0.0221
GO:0033688	Positive regulation of osteoblast proliferation	(red)	1.83	0.0048
GO:0046851	Negative regulation of bone remodeling	(green)	1.83	0.0431
GO:0030199	Collagen fibril organization	(yellow)	1.67	0.00099
GO:0046850	Regulation of bone remodeling	(magenta)	1.51	0.0186
GO:0030514	Negative regulation of bmp signaling pathway	(dark green)	1.51	0.0186
GO:0030510	Regulation of bmp signaling pathway	(light blue)	1.38	0.0065
GO:0061035	Regulation of cartilage development	(gold)	1.37	0.0338
GO:0030500	Regulation of bone mineralization	(purple)	1.34	0.0375
GO:0002062	Chondrocyte differentiation	(dark red)	1.29	0.0455
GO:0030198	Extracellular matrix organization	(grey)	1.21	1.22e-06
GO:0051216	Cartilage development	(black)	1.1	0.0319
GO:0030278	Regulation of ossification	(light green)	1.04	0.0431
GO:1901342	Regulation of vasculature development	(pink)	0.91	0.0370
GO:0048514	Blood vessel morphogenesis	(light grey)	0.9	0.0161

F. PCL_C vs PLA_C

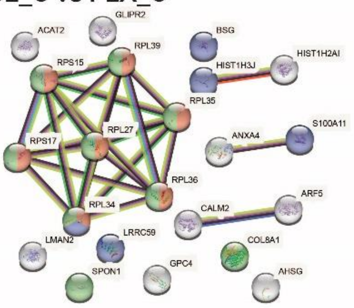

GO-Term	Description	Color	Strength	FDR
GO:0003735	Structural constituent of ribosome	(blue)	1.59	1.37e-06
GO:0045296	Cadherin binding	(red)	1.12	0.0336
GO:0005198	Structural molecular activity	(green)	1.1	2.3e-05

Color Code for Interactions	
(light blue)	from curated databases
(purple)	experimentally determined
(green)	gene neighborhood
(red)	gene fusions
(blue)	gene co-occurence
(yellow-green)	text-mining
(black)	co-expression
(lavender)	protein homology

Figure S6. String protein-protein interaction network with color code and highlighted pathways between materials in chondrogenic medium. Comparisons between PEOT/PBT_C and PCL_C (A), PCL_C and PEOT/PBT_C (B), PEOT/PBT_C and PLA_C (C), PLA_C and PEOT/PBT_C (D), PLA_C and PCL_C (E) and PCL_C and PLA_C (F).

A. PEOT/PBT_O vs PCL_O

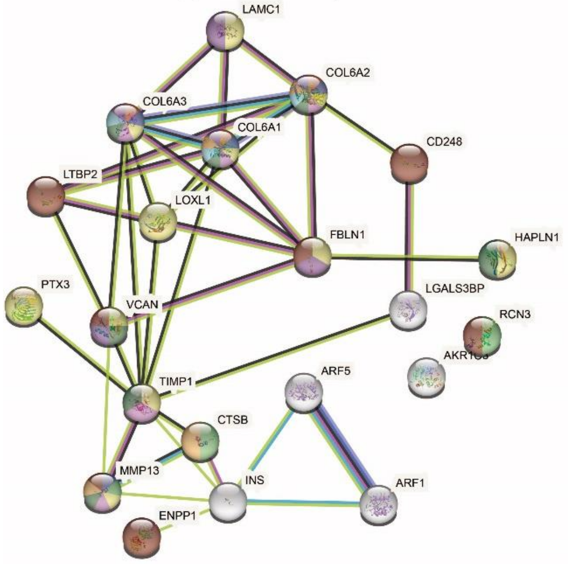

GO-Term	Description	Color	Strength	FDR
GO:0003429	Growth plate cartilage chondrocyte morphogenesis	●	2.22	0.0012
GO:0003417	Growth plate cartilage development	●	2.05	0.0002
GO:0032963	Collagen metabolic process	●	1.65	0.0181
GO:0030198	Extracellular matrix organization	●	1.48	1.9e-10
GO:0051216	Cartilage development	●	1.44	0.0012
GO:0001501	Skeletal system development	●	1.12	0.0008
GO:0030020	ECM structural constituent conferring tensile strength	●	2.0	0.0051
GO:0005518	Collagen binding	●	1.74	0.0016
GO:0005201	Extracellular matrix structural constituent	●	1.67	9.7e-06
GO:0005509	Calcium ion binding	●	0.97	0.0051

B. PEOT/PBT_O vs PLA_O

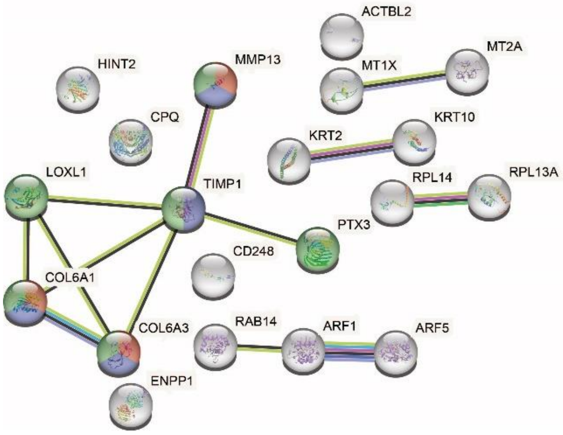

GO-Term	Description	Color	Strength	FDR
GO:0003417	Growth plate cartilage development	●	1.95	0.0273
GO:0051216	Cartilage development	●	1.36	0.0490
GO:0030198	Extracellular matrix organization	●	1.24	0.0114

Color Code for Interactions	
●	from curated databases
●	experimentally determined
●	gene neighborhood
●	gene fusions
●	gene co-occurence
●	text-mining
●	co-expression
●	protein homology

Figure S7. String protein-protein interaction network with color code and highlighted pathways between materials in osteogenic medium. Comparisons between PEOT/PBT_O and PCL_O (A) and PEOT/PBT_O and PLA_O (B).

A. PEOT/PBT _M vs PCL_M

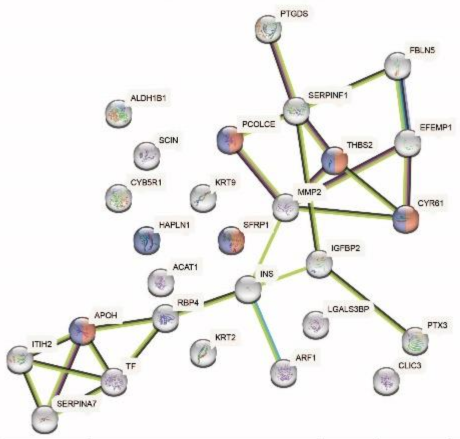

GO-Term	Description	Color	Strength	FDR
GO:0008201	Heparin Binding	blue	1.6	0.0042
GO:0005539	Glycosamminoglycan binding	red	1.22	0.0002

B. PCL_M vs PEOT/PBT_M

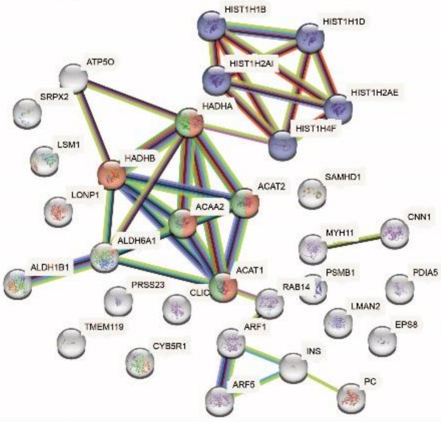

GO-Term	Description	Color	Strength	FDR
GO:0006635	Fatty acid beta-oxidation	blue	1.74	0.0006
GO:0040029	Regulation of gene expression	red	1,18	0,0234
GO:0003985	acetyl-CoA C-acetyltransferase activity	green	2,79	6.60e-07

C. PEOT/PBT_M vs PLA_M

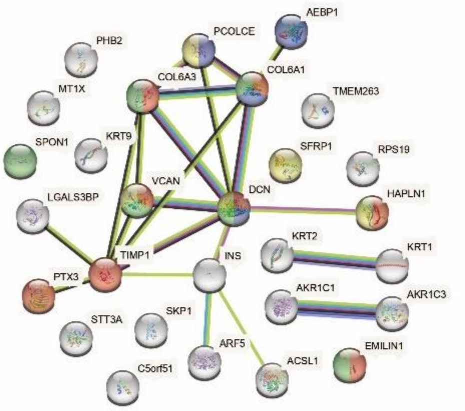

GO-Term	Description	Color	Strength	FDR
GO:0030198	ECM organization	blue	1.22	0.0002
GO:0005518	Collagen binding	red	1.61	0.0035
GO:0005201	ECM structural constituent	green	1.55	3.46e-05
GO:0005539	Glycosaminoglycan binding	yellow	1.16	0.0159

D. PLA_M vs PEOT/PBT_M

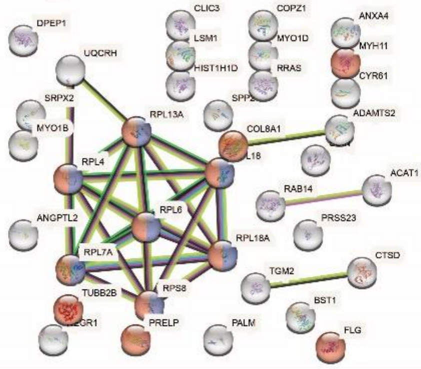

GO-Term	Description	Color	Strength	FDR
GO:0003735	Structural constituent of ribosome	blue	1.38	3.2e-05
GO:0005198	Structural molecule activity	red	1.01	2.9e-06

E. PLA_M vs PCL_M

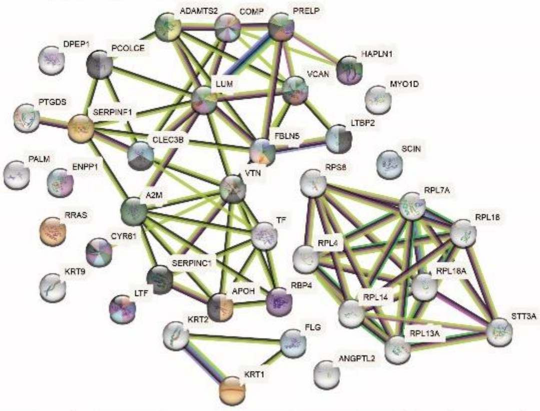

GO-Term	Description	Color	Strength	FDR
GO:0003735	Positive regulation of chondrocyte proliferation	blue	2.3	0.0148
GO:0033690	Positive regulation of osteoblast proliferation	red	2.1	0.0013
GO:0042340	Keratan sulfate catabolic process	green	1.92	0.0429
GO:0030199	Collagen fibril organization	yellow	1.51	3.2e-05
GO:0030500	Regulation of bone mineralization	magenta	1.43	0.0204
GO:0030198	ECM organization	dark green	1.13	2.6e-05
GO:0001503	Ossification	light blue	0.98	0.0262
GO:0045765	Regulation of angiogenesis	ochre	0.92	0.0429
GO:0001501	Skeletal system development	purple	0.91	0.0018
GO:0030021	ECM components for compression resistance	dark red	2.03	0.0018
GO:0005518	Collagen binding	grey	1.47	0.0031
GO:0005539	Glycosaminoglycan binding	black	1.4	1.0e-10
GO:0005201	ECM structural constituent	light green	1.32	0.0018
GO:0005178	Integrin binding	light pink	1.14	0.0365
GO:0005509	Calcium ion binding	light grey	0.76	0.0117

F. PCL_M vs PLA_M

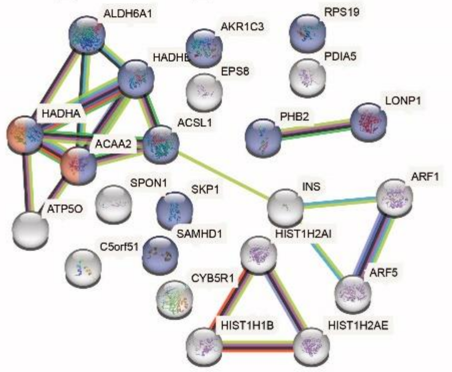

GO-Term	Description	Color	Strength	FDR
GO:0044248	Cellular catabolic process	blue	0.73	0.0197
GO:0003985	Acetyl-CoA activity	red	2.63	0.0213

Color Code for Interactions	
light blue	from curated databases
purple	experimentally determined
green	gene neighborhood
red	gene fusions
blue	gene co-occurence
light green	text-mining
black	co-expression
lavender	protein homology

Figure S8. String protein-protein interaction network with color code and highlighted pathways between materials in maintenance medium. Comparisons between PEOT/PBT_M and PCL_M (A), PCL_M and PEOT/PBT_M (B), PEOT/PBT_M and PLA_M (C), PLA_M and PEOT/PBT_M (D), PLA_M and PCL_M (E) and PCL_M and PLA_M (F).
